# Predicting In-Hospital Mortality in Severe COVID-19: A Systematic Review and External Validation of Clinical Prediction Rules

**DOI:** 10.3390/biomedicines10102414

**Published:** 2022-09-27

**Authors:** Modesto M. Maestre-Muñiz, Ángel Arias, Alfredo J. Lucendo

**Affiliations:** 1 Department of Internal Medicine, Hospital General de Tomelloso, 13700 Ciudad Real, Spain; 2 Department of Medicine and Medical Specialties, Universidad de Alcalá, 28801 Alcalá de Henares, Spain; 3 Hospital General La Mancha Centro, Research Unit, Alcázar de San Juan, 13600 Ciudad Real, Spain; 4 Centro de Investigación Biomédica en Red de Enfermedades Hepáticas y Digestivas (CIBERehd), Instituto de Salud Carlos III, 28006 Madrid, Spain; 5 Instituto de Investigación Sanitaria La Princesa, 28006 Madrid, Spain; 6 Instituto de Investigación Sanitaria de Castilla-La Mancha (IDISCAM), 13700 Tomelloso, Spain; 7 Department of Gastroenterology, Hospital General de Tomelloso, 13700 Ciudad Real, Spain

**Keywords:** COVID-19, clinical prediction rules, nomograms, external validation, systematic review

## Abstract

Multiple prediction models for risk of in-hospital mortality from COVID-19 have been developed, but not applied, to patient cohorts different to those from which they were derived. The MEDLINE, EMBASE, Scopus, and Web of Science (WOS) databases were searched. Risk of bias and applicability were assessed with PROBAST. Nomograms, whose variables were available in a well-defined cohort of 444 patients from our site, were externally validated. Overall, 71 studies, which derived a clinical prediction rule for mortality outcome from COVID-19, were identified. Predictive variables consisted of combinations of patients′ age, chronic conditions, dyspnea/taquipnea, radiographic chest alteration, and analytical values (LDH, CRP, lymphocytes, D-dimer); and markers of respiratory, renal, liver, and myocardial damage, which were mayor predictors in several nomograms. Twenty-five models could be externally validated. Areas under receiver operator curve (AUROC) in predicting mortality ranged from 0.71 to 1 in derivation cohorts; C-index values ranged from 0.823 to 0.970. Overall, 37/71 models provided very-good-to-outstanding test performance. Externally validated nomograms provided lower predictive performances for mortality in their respective derivation cohorts, with the AUROC being 0.654 to 0.806 (poor to acceptable performance). We can conclude that available nomograms were limited in predicting mortality when applied to different populations from which they were derived.

## 1. Introduction

The first cases of pneumonia caused by a new coronavirus [1] were reported just over three years ago in Wuhan, China [2]. The disease caused by coronavirus 2019 (COVID-19) has since spread globally to constitute a public health emergency of international concern [3]. Although most patients had mild or moderate symptoms, a proportion of severely ill patients progressed rapidly to acute respiratory failure, with mortality in 49% [4]. Early identification and supportive care could effectively reduce the incidence of critical illness and in-hospital mortality. Hence, from the early stages of the pandemic, many risk-prediction models, or nomograms, were developed [5] by integrating demographic, clinical, and exploratory findings during early contact with health care systems. However, the merits of most available tools still remain unclear since many were developed to predict a diverse mix of complications (including aggravated disease, need for invasive ventilation, or admission to ICU) in addition to in-hospital mortality; furthermore, most had not been applied to different patient cohorts to those from which they were derived. External validation is essential before implementing nomograms in clinical practice [6]; however, almost no prognostic model for in-hospital mortality for COVID-19 has as yet been validated.

In this research, we aimed to systematically review and critically appraise all currently available prediction models for in-hospital mortality caused by COVID-19. We also aim to compare prediction performances by retrospectively applying nomograms to a well-defined severe patient series admitted to our hospital. 

## 2. Materials and Methods

### 2.1. Eligibility Criteria and Searches

This review was conducted and is reported in accordance with the Preferred Reporting Items for Systematic Reviews and Meta-Analysis (PRISMA) guidelines; a study protocol was registered with PROSPERO [CRD42020226076]. The MEDLINE, EMBASE, Scopus, and Web of Science (WOS) databases were searched for literature published up to 25 August 2021 (Table S1). No restrictions were applied to language or methodological design. No restrictions were placed on prediction horizon (how far ahead the model predicts) within the admission period, or countries or study settings. Additional relevant papers were identified by screening reference lists of included documents. Literature searches were repeated on 20 April 2022 to retrieve the most recent papers and provide updated results.

Studies were included if they (a) described the development/derivation and/or validation of a multivariable tool designed to predict risk of in-hospital mortality in patients with a confirmed diagnosis of severe COVID-19 infection, (b) provided the sensitivity and specificity of the tool or gave sufficient data to allow these metrics to be calculated, and (c) defined the variables or combination of variables used to predict the risk of mortality from COVID-19. 

### 2.2. Study Selection

Two reviewers (MM-M and AJL) independently screened titles and abstracts against eligibility criteria. Potentially eligible papers were obtained, and the full texts were independently examined by the same reviewers. Any disagreements were resolved through discussion.

### 2.3. Data Extraction and Synthesis

Data extraction of included articles was undertaken by two independent reviewers (MM-M and AJL) and checked against full-text papers by a third reviewer (AAA) to ensure accuracy. Using a predeveloped template based on the CHARMS (critical appraisal and data extraction for systematic reviews of prediction modeling studies) checklist [7], information was extracted on study characteristics, source of data, participant eligibility and recruitment method, sample size, method for measurement of outcome, number, type and definition of predictors, number of participants with missing data for each predictor and handling of missing data, modeling method, model performance, and whether priori cut points were used. In addition, we assessed the method used for testing model performance, and the final and other multivariable models. 

For all in-hospital mortality prediction models, area under the receiver operator characteristic curve (AUROC) or concordant (C)-index was used to compare discrimination (the ability of a tool to identify those patients who died from COVID-19 from those who did not). Due to the marked heterogeneity of included studies in terms of the study designs, populations, variable definitions, selection of predictions, use of different tool thresholds, and variable modeling performance, we were unable to perform any meta-analyses. Instead, performance characteristics were summarized in tabular form and a narrative synthesis approach was used. 

### 2.4. Risk of Bias Assessment

PROBAST (prediction model risk of bias assessment tool) was used to assess the risk of bias and applicability of the included studies [8]. PROBAST assesses both the risk of bias and concerns regarding applicability of a study that develops or validates a multivariable diagnostic or prognostic prediction model. It includes 20 signaling questions across 4 domains (participants, predictors, outcome, and analysis). Each domain was rated as having “high”, “low”, or “unclear” (where insufficient information was provided) risk of bias. Two reviewers (A.A. and A.J.L.) independently assessed each study. Ratings were compared and disagreements were resolved by consensus.

### 2.5. External Validation of Included Clinical Prediction Rules

External validation was carried out as long as the variables integrated in a model were available among those registered in an external validation cohort of patients. No other restrictions were placed on the type of variable that could be included in a tool. 

Data on 444 adults with confirmed SARS-CoV-2 infections, who were admitted to our hospital due to severe COVID-19 between 26 February to 31 May 2020 and with a 90-day follow-up period available, were used for the external validation of selected clinical prediction rules. Detailed methods and clinical and demographic characteristics of this patient series have been previously described [9,10]. All patients were independent from the data used in the derivation of any of the included clinical prediction rules. Data from the validation cohort were recoded to reproduce predictors and primary outcomes of each included clinical prediction rule, and modifications were made to match available data. The same point assignment and cut-off values provided by original derivation cohorts were used for external validation analyses.

## 3. Results

**Figure 1:**
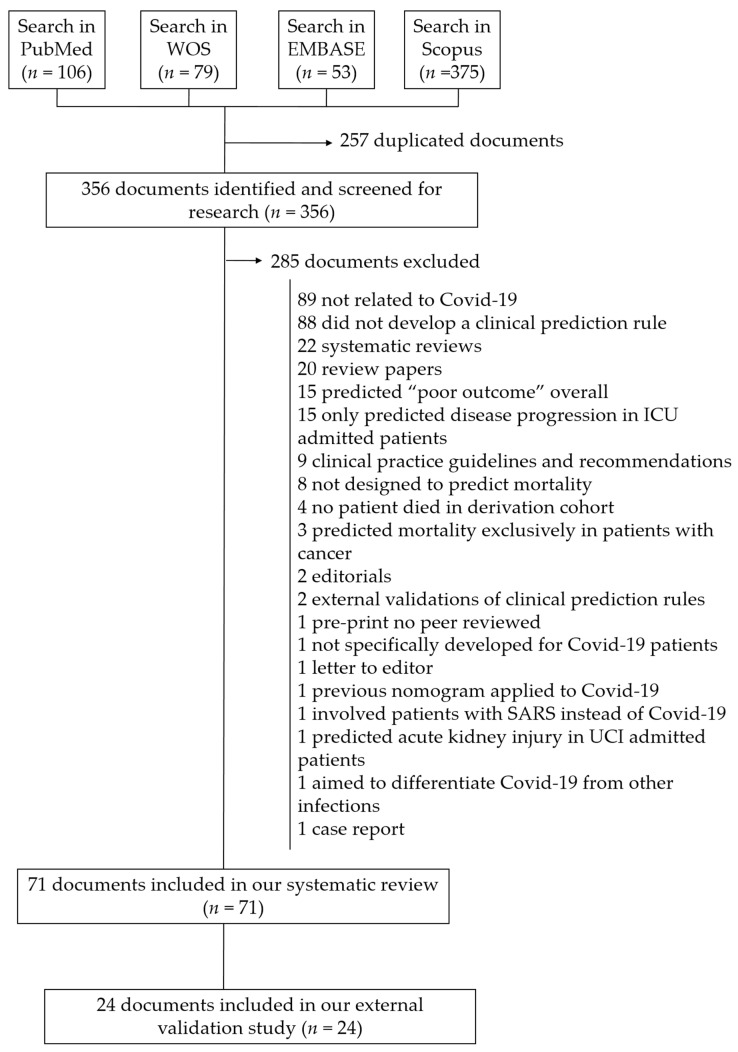
Flow diagram of included documents.

The systematic review flow-chart is shown in Figure 1. Overall, 71 studies with a derivation of a clinical prediction rule for mortality outcome from COVID-19 were identified [2,11,12,13,14,15,16,17,18,19,20,21,22,23,24,25,26,27,28,29,30,31,32,33,34,35,36,37,38,39,40,41,42,43,44,45,46,47,48,49,50,51,52,53,54,55,56,57,58,59,60,61,62,63,64,65,66,67,68,69,70,71,72,73,74,75,76,77,78,79,80]. Forty studies used data from patients from China [2,11,12,13,14,20,21,22,24,26,27,29,30,31,32,33,40,41,42,44,45,48,51,53,54,55,59,60,61,63,65,66,67,69,70,74,78,79,80], eight from the United States [37,38,43,46,62,64,75,77], five from Spain [28,35,36,50,76], three from the United Kingdom [15,18,23], six from Italy [17,47,52,57,58,73], two from Mexico [16,49], four from Korea [19,34,68,71], two from Turkey [39,56], and one from Pakistan [25]. 

The search strategy in the different databases is detailed in Table S1. The characteristics of the included clinical prediction rules are shown in Table S2.

Study data were collected between 1 January and 20 May 2020, during the first wave of the pandemic; the earliest data were provided by Chinese hospitals, all of them prior to 31 March 2020. The latest admissions were recorded in hospitals in the US and Mexico, all of them between the beginning of March and the end of May 2020. 

Fifty-five studies aimed to exclusively predict in-hospital mortality [2,11,14,15,16,18,19,20,21,22,23,24,29,30,31,32,34,35,36,39,40,41,42,43,44,45,47,50,51,52,54,56,57,58,59,60,61,62,64,65,66,67,68,69,70,71,72,73,74,75,76,77,79], while thirteen reported on combined outcomes (progression of COVID-19 to severe, including need for invasive artificial respiration, ICU admission, or death) [12,17,25,26,27,28,33,35,37,38,46,55,80]. We ensured deceased patients were effectively included among the dataset and numbers reported. Among studies aimed at reporting on disease progression, a predictive nomogram for mortality was specifically provided in nine of the studies [17,25,27,28,37,38,46,49,80] but not in the other four [12,26,33,55]. 

Study setting included general population from database sources [19,23,49,58,68,71], clinical records from series of patients admitted to hospital [2,11,12,14,16,17,18,20,21,22,24,25,27,28,29,30,31,32,34,35,36,37,38,39,40,41,42,43,44,45,46,47,48,50,51,52,53,54,55,56,57,59,60,61,62,63,64,65,66,67,69,70,72,73,75,77,79,80], and healthcare databases [15,74,76]. One clinical prediction rule was developed exclusively from patients admitted to ICU [33]. 

All studies used a retrospective design for derivation and 21 were performed in multiple centers [12,13,15,16,19,21,23,24,26,28,29,31,34,43,45,47,48,49,52,54]. Among nomograms developed from hospital records, the derivation sample size was small (less than 300 patients) in 24 studies [2,12,14,17,25,27,28,30,31,32,33,34,35,37,40,42,47,48,55,67,73,78,79] and relatively small (300 to 500 patients) in 14 studies [11,13,16,18,20,22,39,41,44,51,60,74,75,80]. Only 20 hospital-based studies derived mortality prediction rules from series over 1000 patients [21,24,26,36,38,43,50,52,54,59,62,63,65,66,68,69,70,71,72,77]. Large administrative databases of COVID-19-infected patients and hospitalizations of over 10,000 patients were used in four studies [15,19,23,49] (Table S2). 

Thirty-four nomograms were derived and validated (in different patients from the same institution) in the same study; random training test splits [14,17,18,19,23,24,30,32,33,41,45,50,51,55,59,60,61,62,63,65,66,67,71,75,76,77] and temporal splits [15,43,44,46,53,70] were performed in fourteen and five studies, respectively. One study performed validation by leave-one-hospital-out cross validation [72].

Only 12 studies externally validated their clinical prediction tool in a cohort of patients in a different institution [11,13,26,38,48,52,55,65,66,70,74,75] from which it was derived.

Overall, data from 317,840 SARS-Cov-2-infected patients were included in the derivation cohorts of the 71 predictive models, 36,882 of whom died. The percentage of deaths varied widely between studies, from 2.2% to 48.9%.

Patients with missing data for any predictor were excluded from analyses in 17 studies [16,18,19,24,29,30,32,40,42,48,51,53,56,59,64,71,78], missing values were provided by imputation methods in a further 21 [11,14,23,26,33,38,43,44,45,50,52,58,60,63,69,72,74,75], or were not reported in the remaining [2,12,13,15,17,20,21,22,25,28,31,34,35,36,37,39,41,46,47,49,54,55,57,61,66,67,73,76,77,79,80]. The absence of predictors was most likely a random event and was not associated with the outcome. 

### 3.1. Models to Predict Risks of COVID-19-Related Mortality in Hospitalized Patients

Overall, 67 models identified predictors for risk of COVID-19-related mortality in patients admitted to hospitals, and a further 3 also included information from the general population and/or out-patients [19,23,49,71,77]. The vast majority of these studies used multivariable logistic regression models to identify predictors [2,12,13,14,16,17,18,20,21,22,24,25,26,27,30,31,32,33,34,35,37,38,39,41,43,45,46,47,48,49,51,52,53,54,56,57,58,59,62,63,64,65,66,68,69,70,71,72,73,74,75,76,77,78,79,80]. Least absolute shrinkage and selection operator (LASSO) were used in 14 studies [11,19,20,23,26,38,42,51,55,59,61,63,67,79], machine learning techniques were applied in an additional 12 [15,19,24,33,38,43,44,48,50,74,75,76], random decision forest or gradient decision trees (GBDT) in 10 studies [24,29,43,48,50,60,63,72,75,76], and artificial neural networks in 4 studies [18,36,50,76]. In nineteen studies, several of these methods of derivation were applied together [18,19,20,24,26,33,38,43,48,50,51,59,60,63,72,74,75,76,79]. 

The complete data on predictors were reported in all the 71 studies, and formulas to calculate mortality risks were provided or could be extracted in 32 studies (Table S2). The authors of three additional studies for which we were unable to find the formula [2,31,32] were twice contacted by email but did not respond. In the remaining studies, the formula could not be provided, as these predictive models were derived from complex techniques (decision trees or machine learning). Eight predictive models provided an online-available tool to automatically predict outcomes [15,16,17,26,48,50,52].

The most frequently used prognostic variables for mortality (included at least five times among the different nomograms) were age (in 53 models), diabetes mellitus (in 11 models), chronic lung disease (COPD or asthma) (8 models), heart disease or cardiac failure (in 13), chronic kidney insufficiency (in 10), hypertension (in 7), and chronic liver disease (in 5 models). Comorbidity, defined either as number of conditions selected from a predefined list or Charlons index, were recognized as determinants for mortality in nine additional models. 

Clinical predictors at admission included in final models consisted of dyspnea/taquipnea (14 models) and radiographic chest alteration (7 models). Analytical variables at admission identified as predictors included serum lactate dehydrogenase (LDH) in 24 prediction rules, C-reactive protein in 28 models, lymphocytes (either absolute number per µL or neutrophils-to-lymphocytes ratio) in 23 models, renal function (defined in terms of urea, BUN, serum creatinine, or glomerular filtration rates) in 20 models, and respiratory function parameters (peripheral O2 saturation, supplemental O2 at admission, PaO2/FiO2, or alveolar-arterial oxygen gradient) in 23 prediction models. D-dimer was included in 16 models and platelet count in 8 additional ones. Markers for liver injury (elevated billirrubin or aminotransferase levels) and myocardial damage (including either troponine I, myoglobine, or creatine phosphokinase) were included as predictors for mortality in 17 and 7 nomograms, respectively. Details on all predictors included in final models are provided in Table S2.

### 3.2. Risk of Bias

**Figure 2:**
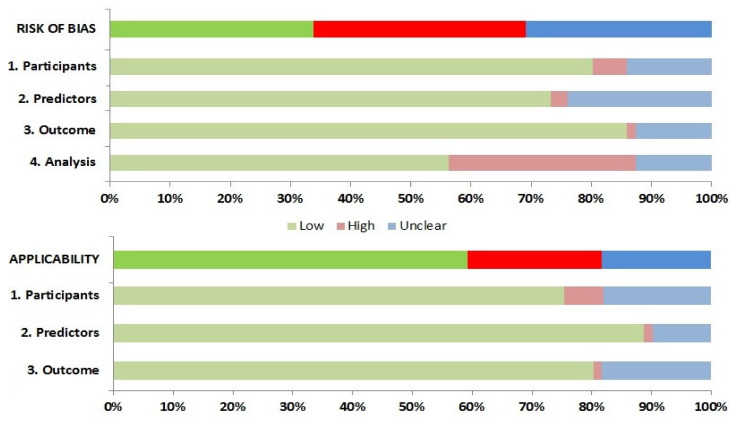
Risk of bias assessment according to the Prediction model Risk of Bias Assessment Tool (PROBAST).

Twenty-five studies were at high risk of bias (ROB) according to assessment with PROBAST, and a further twenty-three showed an unclear ROB (Figure 2 and Table S3). This suggests that their performances when used in predicting in-hospital mortality caused by COVID-19 is probably lower than that reported. Fifty-seven studies (80.3%) were evaluated as being of low ROB for the participants′ domain, thus indicating that the participants enrolled in the studies were representative of the models′ targeted populations. All but 19 studies had a low ROB for the predictor domain (with the remaining being unclear), which indicates that predictors were available at the models’ intended time of use and clearly defined, or independent from mortality. There were concerns about bias induced by the outcome measurement in ten studies, especially due to lack of information on time intervals between predictor assessment and outcome determination as a result of registering data from infected outpatients, or for confusingly considering losses to follow-up as deceased patients. Twenty-two studies were evaluated as high ROB for the analysis domain, mainly because calibration was not assessed, or due to risk of model overfitting when complex modeling strategies were used. 

The applicability of the different CPRs was also assessed. High ROB in population domain was mainly due to inclusion of patients without severe COVID-19 in original derivation cohorts. Studies that derived CPR for disease progression outcomes were evaluated as being of unclear ROB. Predictors derived from patient cohorts with small numbers of deceased patients determined their unclear applicability. Predictors obtained from patients in ICU were considered to be of high ROB regarding their applicability to our systematic review. Combined outcomes (e.g., disease progression) were considered of high or unclear ROB when only a minority of deceased patients was included in derivation cohorts.

### 3.3. Evaluation of Tool Performance in Predicting COVID-19-Related Mortality

Studies that predicted mortality in derivation cohorts reported AUROC between 0.701 and 1. C-index values ranged between 0.823 and 0.970. These values were over 0.9 (very good test) and over 0.97 (outstanding test) in 31 and 6 of the 71 predictive models, respectively. When provided, sensitivity values for cut-off points provided by the different authors in derivation cohorts ranged between 32% and 98.4%, with specificity ranging from to 100% to 38.6%. 

Overall, 33 predictive models were applied to new patients (internal validation). No major differences among derivation and internal validation cohorts were found in 5 of the studies [11,23,43,51,66]; in 7 studies, a significantly lower mortality rate was documented in internal validation cohorts [17,38,44,65,70,81,82], and 14 studies provided no details on differences in demographic and clinical features between derivation and internal validation patient subsets [14,18,19,24,26,30,32,33,45,46,48,50,53,55]. Overall, prediction models provided AUROC ranging from 0.73 to 0.991 and were quite similar to those provided in derivation cohorts, with only two nomograms proving lower predictive performance [17,30].

For all the 11 studies that externally validated their clinical prediction rules in patients from different institutions, the observed prediction performances were quite similar in each validation subcohort compared to respective derivation cohorts [11,13,26,38,48,52,55,65,70,74,75]. Thus, AUROC in Weng et al. were 0.921 and 0.975 in derivation and external validation cohorts [11], respectively; Xiao et al. studies provided 0.943 and 0.826 for derivation and validation, respectively [81]; Liang et al. provided AUROC of 0.88 for both derivation and validation cohorts [26]; the XGBoost model used by Vaid et al. provided AUC-ROC values 0.853 and 0.85, respectively, for derivation and validation cohorts [38]; Magro et al. provided AUROC values of 0.822 and 0.820 for the derivation and external validation cohorts [52]; and they were 0.943 and 0.878 in the study by Zhang et al. [55]. In addition, Li developed a CPR from a hospital in Wuhan, China, which provided a C-index of 0.97 in the derivation cohort. It was 0.96 in the internal validation cohort recruited in a second hospital in Wuhan and 0.92 when externally validated in a third neighboring hospital [70]. Similar results were found by He et al. as well in their study involving patients from three hospitals in the Hubei province, China [65]. 

Notably, external validation was mostly performed for patient cohorts from the same cities or countries that the prediction rules were derived in. However, Rahman et al. derived a prognostic model from 375 COVID-19 patients admitted to Tongji Hospital, China, with an AUROC value of 0.961; when it was validated with an external cohort of 103 patients of Dhaka Medical College, Bangladesh, the AUC value was 0.963 [75].

Sample sizes for data that were used in external validation of each clinical prediction rule were smaller than the respective derivation cohort.

### 3.4. External Validation in the Same New Cohort of Patients

**Figure 3:**
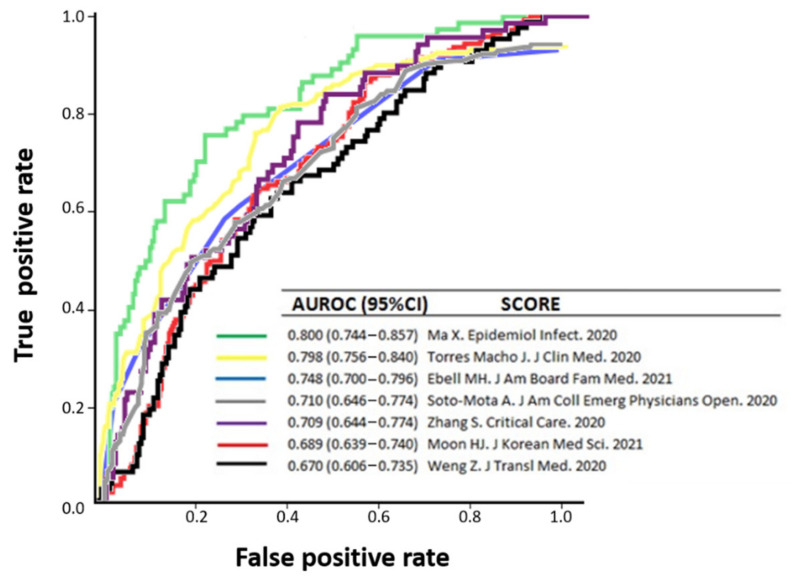
Comparison of receiver operator characteristic curves for low-risk-of-bias-predictive models for in-hospital mortality [11,16,30,45,50,62,71], applicable to our external validation population.

Predicting variables included in 25 out of the 71 prognostic models identified in our systematic review were present in our local cohort of patients [11,12,15,16,22,25,26,28,30,34,35,37,39,40,45,47,48,49,50,56,60,62,71,81], thus allowing external validation on its performance in a separate clinical setting [9] to assess how the rule could be used in real-life. Prediction rules were validated on subcohorts that included 208 to 444 patients (depending on the number of cases that had all the variables incorporated in each model). For all 25 prognostic models, prediction performances for mortality were notably lower than in the respective derivation cohorts or internal validation cohorts, with AUROC values ranging from 0.654 to 0.806 (poor to acceptable performance). No nomogram was considered good or outstanding to predict in-hospital mortality among our own cohort of patients. A wide variability of sensitivity (ranging 15.5% to 100%) and specificity (1.3% to 98.5%) was found when the best cut-off values (as provided by original authors) were selected (Table 1). Figure 3 represents a comparator of receiver operator characteristic curves for low ROB predictive models for in-hospital mortality, applicable to our external validation population.

## 4. Discussion

In this systematic review, we identified, retrieved, and critically appraised 71 individual studies that develop prediction models to support the prognostication of death among patients with COVID-19. To our knowledge, this is the first systematic assessment and comparison of prognostic performances of existing clinical prediction rules on risk for in-hospital mortality caused by severe COVID-19. All models were developed during the first wave of the pandemic and reported very-good-to-outstanding predictive performances in derivation and internal validation cohorts. 

Predictive tools comprised simple analytical values-based nomograms, nomograms which included symptoms, analytical values and imaging tests, and more complex diagnostic prediction models that incorporated symptoms, test results, and comorbid conditions. Predicting factors included in the different nomograms varied widely among studies, but many have been repeatedly associated with poor prognosis in COVID-19. Thus, advanced age, COPD, heart disease, hypertension, chronic kidney failure, and diabetes were positively associated with risk of death in at least five nomograms and have been related to progression to severe disease as well [83]. In contrast, male sex, smoking history, and obesity were exceptionally included in nomograms, despite being identified as risk factors for progression to severe disease and death in COVID-19 patients in some studies [84,85,86]. Organ failure, including pneumonia, respiratory insufficiency, and ischemic cardiac or liver injury were repeatedly included in nomograms and have been related to poor prognosis in independent research [87,88,89]. Inflammatory markers such as C-reactive protein, D-dimer, as well as lymphopenia, thrombocytopenia, and elevated LHD were recognized by the earlier literature on COVID-19 [90] and the most recent research [9]. Models developed using data from different countries agreed on including common predictive analytical values, despite most nomograms being developed by China. 

The methods to develop the different prognostic models available varied greatly in terms of modeling technique, methodology, and rigor of construction. Only 15 were assessed as of low ROB for development and applicability together. The prognostic performances of most tools were evaluated solely within the study datasets, with internal validation carried out on a subset of the original cohorts, thus reproducing the AUROC values provided in derivation subsets. Internally validating on the same cohort used for derivation usually overestimated the performance of scores [91]. For relatively small data sets, as those used to derive most of the nomograms, internal validation by bootstrap techniques might not be sufficient or indicative for the model′s performance in future patients [6], despite demonstrating the stability and quality of the predictors selected within the same cohort. Only 11 tools were externally validated in other participants in the same article. However, these were almost always on patients from the same or neighboring cities as those included in the derivation cohort and therefore likely to have similar characteristics, thus showing overlapping results. 

Until now, one single nomogram had been externally evaluated for its predictive capacity for COVID-19-related in-hospital mortality in a different series [49]. The predictive performance of this nomogram developed in Mexico, reduced markedly when applied to a different patient dataset from the same country [16], with the AUROC decreasing from 0.823 to 0.690. A still unpublished prediction rule derived from patients in Wuhan, China, provided C-index for death of 0.91 that only decreased to 0.74 when it was externally validated in patients admitted to hospital in London (UK) [92]. This research is, to our knowledge, the first in externally validating prediction rules for in-hospital mortality caused by COVID-19 altogether and provides further evidence of their limited performance when applied to different clinical settings. The study by Rahman et al. [75] represents a second attempt to externally validate; better results were produced, but these were affected by high risks of bias. It can be suggested therefore that each nomogram developed to predict mortality from COVID-19 should be applied to the same clinical settings from where it was derived. 

The main strengths of this study include its systematic search on the multiple literature databases that index the main results of research on COVID-19; the fact that the research is up to date; and the critical appraisal of the methods and ROB of the studies retrieved. The different nomograms have been analyzed in detail and formulas to allow for the estimating of mortality risk for any population by using each of them were provided whenever possible. Finally, the predictive performance of nomograms based on demographic, clinical, and analytical parameters available in usual clinical practice were evaluated in a single external series of COVID-19 patients admitted to hospital.

Some weaknesses should be acknowledged for our study. These are mainly as a result of the heterogeneity of source documents, which derive from different populations with variable disease severity, and cared for in not necessarily comparable healthcare settings. While certain clinical and laboratory variables were identified to contribute objectively to mortality in most studies, many others varied widely among different prediction models. As several nomograms included variables that are not routinely used in clinical practice, we could not provide external validation. Additionally, we did not retrieve studies only available as preprints, which might improve after peer review. Finally, no prediction model was derived from or validated in patients infected by COVID-19 during the second and successive waves of the pandemic, and their current usefulness has not been evaluated. 

## 5. Conclusions

To conclude, our research demonstrates the limitations of prognostic rules for risk of mortality from COVID-19 when applied to different populations from which they were derived. Demographic, clinical, and analytical determinants for risk of mortality are influenced and modulated by many factors inherent to each clinical setting, which are not easily controllable or reproducible. Once main determinants for COVID-19-related mortality at hospital admission have been identified, the best predictive models could be those developed in each particular clinical setting.

## Figures and Tables

**Table 1:** Prognostic models for mortality from COVID-19, externally validated in a cohort of patients admitted to Tomelloso General Hospital, Spain. Risk categories and cut-off points were defined according to original models. Performance of each model (area under receiver operator curve (AUROC), sensitivity (%), specificity (%), and positive predictive value (PPV)/negative predictive value (NPV) (%) with 95% confidence intervals), if reported, were calculated over model validation subcohort of N participants.

Clinical Prediction Tool; Author, Journal, Year Reference	N	Mean Score (Standard Deviation; Rank)	Risk Categories		AUROC (95% CI)	Cut-Off Point	Sensitivity (95% CI)	Specificity (95% CI)	PPV (95% CI)	NPV (95% CI)
Acar HC. BMC Infect Dis 2021 [56]	84	21.1 (8.6; 4 to 42)	Low risk	27 (32.1%)	0.806 (0.710–0.901)	<17 points >38 points	100% 12.5%	28.1% 98.5%	39.7% 66.7%	100% 82.7%
			High risk	3 (3.6%)						
Bello-Chavolla OY. J Clin Endocrinol Metab 2020 [49]	444	9.85 (3.1; 6 to 16)	Low risk	4 (0.9%)	0.672 (0.620–0.724)	≤0 points	100% (97.4–100)	1.3% (0.5–3.4)	32.3% (28.1–36.8)	100% (51–100)
			Mild risk	19 (4.3%)		≤3 points	98.6% (95–99.6)	7% (4.6–10.4)	33.3% (28.9–37.9)	91.3% (73.2–97.6)
			Moderate risk	17 (3.8%)		≤6 points	95.1% (90.2–97.6)	10.9% (7.9–14.9)	33.4% (29–38.2)	82.5% (68–91.3)
			High risk	111 (25%)		≥10 points	83.8% (76.9–89)	42.4% (36.9–48)	40.6% (35.1–46.3)	84.8% (78.2–89.6)
			Very high risk	293 (66%)						
Cheng A. Int J Antimicrob Agents 2020 [22]	328	7.07 (5.7; 1.39 to 37.28)	Low risk	238 (72.8%)	0.654 (0.588–0.720)	BUN ≥ 4.6 and D-Dimer ≥ 0.845	46.1% (36.1–56.4)	79.8% (74.3–84.4)	46.1% (36.1–56.4)	79.8% (74.3–84.4)
			High risk	89 (27.2%)						
Chowdhury MHE. Cognit Comput 2021 [60]			Low risk	3 (1.1%)	0.798 (0.736–0.859)	<10.4 pts	100% (94.9–100)	1.9% (0.7–4.7)	25.2% (20.5–30.6)	100% (51–100)
	286	15.13 (2.2; 9 to 22.3)	Moderate risk	28 (9.8%)		10.4–12.65				
			High risk	254 (89.1%)		>12.65 pts	97.2% (90.3–99.2)	14% (10–19.2)	27.2% (22.1–32.9)	93.8 (79.9–98.3)
Ebell MH. J Am Board Fam Med 2021 [62]	438	NoLab: 5.76 (2; 3 to 10)	Low risk	0	0.748 (0.700–0.796)	0–1 pts	−	−	−	−
			Moderate risk	256 (58.4%)		2–5 pts	65.9% (57.7–73.3)	69.7% (64.2–74.6)	50% (42.8–57.2)	81.6% (76.4–85.9)
			High risk	182 (41.6%)		≥6 pts				
	420	SimpleLab: 14.3 (5.4; 6 to 29)	Low risk	33 (7.9%)	0.752 (0.702–0.803)	0–7 pts	98.5% (94.6–99.6)	10.7% (7.6–14.8)	33.1% (28.6–37.9	93.9 (80.4–98.3)
			Moderate risk	108 (25.7%)		8–11 pts	89.2% (82.7–93.5)	43.8% (38.2–49.5)	41.6% (35.9–47.4)	90.1% (84–94)
			High risk	279 (66.4%)		≥12 pts				
Hu C. Int J Epidemiol 2020 [48]	324	Risk Score −1.71 (2.1; −19.7 to 2.5)	>50%	278 (85.8%)	0.679 (0.614–0.745)	50%	25.3% (17.3–35.3)	89.9% (85.4–93.1)	47.8% (34.1–61.9)	76.6% (71.3–81.2)
		Probability of death 24.2 (21; 0 to 92.5)	<50%	46 (14.2%)						
Ji D. Clin Infect Dis. 2020 [12]	334	10.88 (2; 5 to 13)	Low risk	15 (4.5%)	0.670 (0.608–0.732)	6	100% (95.5–100)	5.6% (3.4–9.1)	25.7% (21.2–30.8)	100% (78.5–100)
			Intermediate risk	41 (12.3%)						
			High risk	278 (83.2%)		9	98.8% (93.4–99.8)	21.5% (16.9–27)	29.1% (24.1–34.7)	98.2% (90.4–99.7)
Kamran SM. Cureos. 2020 [25]	358	6.58 (1.5; 3 to 10)	Low risk	304 (84.9%)	0.756 (0.702–0.809)	9 points	32.7% (24.7–41.9)	92.7% 88.8–95.4)	66.7% (53.4–77.8)	75.7% (70.5–80.1)
			High risk	54 (15.1%)						
			Low risk	56 (13.4%)	0.765 (0.717–0.813)	≤3 points	97.7% (93.4–99.2	18.4% (14.4–23.3)	34.9% (30.2–40)	94.6% (85.4–98.2)
Knight SR. BMJ. 2020 [15]	417	8.60 (4; 0 to 18)	Moderate risk	131 (31.4%)		≤8 points	79.8% (72.1–85.9)	55.9% (50.1–61.5)	44.8% (38.5–51.2)	86.1% (80.4–90.3)
			High risk	230 (51.2%)		≤14 points	12.4% (7.8–19.2)	96.9% (94.2–98.3)	64% (44.5–79.8)	71.2% (66.5–75.4)
Liang W. JAMA Intern Med 2020 [26]	304	162.8 (37.2; 70.5 to 268)	−	−	0.795 (0.739–0.850)		−	−	−	−
Lorente L. Anaesth Crit Care Pain Med 2020 [28]	438	14.69 (2; 121 to 28.6)	Low risk	58 (123.2%)	0.636 (0.582–0.689)	>13%	95% (90–97.5)	17.1% (13.2–21.7)	34.7% (30.1–39.7)	87.9% (77.1–94)
			High risk	380 (86.8%)						
Ma X. Epidemiol Infect 2020 [30]	326	0.4148 (2.4; −6.41 to 8.89)	−	−	0.800 (0.744–0.857)	−	−	−	−	−
Moon HJ. J Korean Med Sci. 2021 [71]	444	LR: 196.3 (41.2; 59 to 271.5)			0.689 (0.639–0.740)		−	−	−	−
	444	CR: 186.4 (37.3; 60.6 to 255.2)			0.688 (0.638–0.739)		−	−	−	−
Park JG. BMJ Open. 2020 [34]	331	3.24 (5.8; 0.18 to 89.01)	Low risk	293 (88.5%)	0.725 (0.667–0.782)	≥4.95	22.3% (15.1–31.8)	92.8% (88.8–95.5)	55.3% (39.7–69.9)	75.1% (69.8–79.7)
			High risk	38 (11.5%)						
Salto-Alejandre S. J Infection. 2020 [35]	321	−0.87 (1.98; −4.6 to 3.1)	Low risk	234 (72.9%)	0.654 (0.587–0.720)	>0.5	38.5% (28.4–49.6)	76.5% (70.8–81.4)	34.5% (25.3–44.9)	79.5% (73.9–84.2)
			High risk	87 (27.1%)						
Soto-Mota A et al. J Am Coll Emerg Physicians Open 2020 [16]	298	55.79 (32.3; 0 to 100)	>65	153 (51.3%)	0.710 (0.646–0.774)	>65	68.3% (57.6–77.4)	41.2% (34.8–47.5)	30.6% (24.4–37.6)	77.4% (68.9–84.1)
			<65	145 (48.7%)						
Torres-Macho J et al. J Clin Med. 2020 [50]	444	265.3 (46.9; 124 to 391)	−	−	0.798 (0.756–0.840)	−	−	−	−	−
Turcotte JJ. PLoS One. 2020 [40]	426	15.4 (1.7; 12.8 to 20.5)	−	−	0.672 (0.618–0.726)	-	−	−	−	−
Varol Y. Int J Clin Pract. 2020 [39]	438	3.9 (1.2; 0 to 5)	Low risk	59 (13.5%)	0.703 (0.653–0.753	>2.5	98.6% (94.9–99.6)	19.1% (15–23.9)	36.1% (31.5–41.1)	96.6% (88.5–99.1)
			High risk	379 (86.5%)						
Wang J. J Int Med Res. 2020 [14]	208	83.1 (18.5; 23.3 to 167.3)	Low risk	108 (51.9%)	0.732 (0.651–0.813)	85	78% (63.3–88)	40.7% (33.6–48.3)	24.4% (17.9–32.4)	88.3% (79.3–93.7)
			High risk	100 (48.1%)						
Weng Z. J Transl Med 2020 [11]	320	90.28 (34; 12.1 to 223.9)	Low risk	44 (13.8%)	0.670 (0.606–0.735)	<59	95.3% (88.6–98.2)	17.1% (12.8–22.4)	29.7% (24.6–35.4)	90.9% (78.8–96.4)
			Moderate risk	193 (60.3%)						
			High risk	83 (25.9%)		>101	44.2% (34.2–54.7)	80.8% (75.2–85.3)	45.8% (35.5–56.5)	79.7% (74.2–84.4)
Xiao LS. EBioMedicine 2020 [13]	321	0.145 (1.3; −2.60 to 2.06)	Low risk	47 (14.6%)	0.562 (0.493–0.632)	≥−1.508	90.9% (82.4–95.5)	83.6% (78.4–87.7)	63.6% (54.3–72)	96.7% (93.3–98.4)
			High risk	274 (85.4%)						
Zhang S. Critical Care 2020 [45]	305	146.8 (32.7; 19.3 to 202.5)	−	−	0.709 (0.644–0.774)	−	−	−	−	−
Zinellu A. Eur J Clin Invest 2020 [47]	331	1.44 (0.66; 0.28 to 5.88)	Low risk	212 (64%)	0.670 (0.607–0.733)	>1.49	54.3% (44.2–64)	71.3% (65.2–76.7)	42.9% (34.3–51.8)	79.7% (73.8–84.6)
			High risk	119 (36%)						

## Data Availability

The data that support the findings of external validation of clinical prediction rules for this study are available from the corresponding author upon reasonable request.

## Supplementary Materials

The following supporting information can be downloaded at: https://www.mdpi.com/article/10.3390/biomedicines10102414/s1. Table S1: Search strategies carried out in four bibliographic databases of documents that report on clinical prediction models for hospital mortality caused by COVID-19. Table S2: Overview of prediction models for mortality risk from COVID-19 identified in a systematic review of the literature, and performance of each model in derivation cohorts. Formulas to calculate mortality risk to be applied to any population were provided or extracted from original documents. Table S3: Risk of bias assessment (using PROBAST) based on four domains across the studies that developed and/or validated prediction models for in-hospital mortality due to coronavirus disease 2019.

## References

[1] Lu R., Zhao X., Li J., Niu P., Yang B., Wu H., Wang W., Song H., Huang B., Zhu N. (2020). Genomic Characterisation and Epidemiology of 2019 Novel Coronavirus: Implications for Virus Origins and Receptor Binding. Lancet.

[2] Zhou J., Huang L., Chen J., Yuan X., Shen Q., Dong S., Cheng B., Guo T.-M. (2020). Clinical Features Predicting Mortality Risk in Older Patients with COVID-19. Curr. Med. Res. Opin..

[3] Phelan A.L., Katz R., Gostin L.O. (2020). The Novel Coronavirus Originating in Wuhan, China: Challenges for Global Health Governance. JAMA.

[4] Wu Z., McGoogan J.M. (2020). Characteristics of and Important Lessons from the Coronavirus Disease 2019 (COVID-19) Outbreak in China: Summary of a Report of 72,314 Cases from the Chinese Center for Disease Control and Prevention. JAMA.

[5] Wynants L., Van Calster B., Collins G.S., Riley R.D., Heinze G., Schuit E., Bonten M.M.J., Dahly D.L., Damen J.A.A., Debray T.P.A. (2020). Prediction Models for Diagnosis and Prognosis of COVID-19: Systematic Review and Critical Appraisal. BMJ.

[6] Bleeker S.E., Moll H.A., Steyerberg E.W., Donders A.R.T., Derksen-Lubsen G., Grobbee D.E., Moons K.G.M. (2003). External Validation Is Necessary in Prediction Research: A Clinical Example. J. Clin. Epidemiol..

[7] Moons K.G.M., de Groot J.A.H., Bouwmeester W., Vergouwe Y., Mallett S., Altman D.G., Reitsma J.B., Collins G.S. (2014). Critical Appraisal and Data Extraction for Systematic Reviews of Prediction Modelling Studies: The CHARMS Checklist. PLoS Med..

[8] Moons K.G.M., Wolff R.F., Riley R.D., Whiting P.F., Westwood M., Collins G.S., Reitsma J.B., Kleijnen J., Mallett S. (2019). PROBAST: A Tool to Assess Risk of Bias and Applicability of Prediction Model Studies: Explanation and Elaboration. Ann. Intern. Med..

[9] Maestre-Muñiz M.M., Arias Á., Arias-González L., Angulo-Lara B., Lucendo A.J. (2021). Prognostic Factors at Admission for In-Hospital Mortality from COVID-19 Infection in an Older Rural Population in Central Spain. J. Clin. Med..

[10] Maestre-Muñiz M.M., Arias Á., Mata-Vázquez E., Martín-Toledano M., López-Larramona G., Ruiz-Chicote A.M., Nieto-Sandoval B., Lucendo A.J. (2021). Long-Term Outcomes of Patients with Coronavirus Disease 2019 at One Year after Hospital Discharge. J. Clin. Med..

[11] Weng Z., Chen Q., Li S., Li H., Zhang Q., Lu S., Wu L., Xiong L., Mi B., Liu D. (2020). ANDC: An Early Warning Score to Predict Mortality Risk for Patients with Coronavirus Disease 2019. J. Transl. Med..

[12] Ji D., Zhang D., Xu J., Chen Z., Yang T., Zhao P., Chen G., Cheng G., Wang Y., Bi J. (2020). Prediction for Progression Risk in Patients with COVID-19 Pneumonia: The CALL Score. Clin. Infect. Dis..

[13] Xiao L., Zhang W.-F., Gong M., Zhang Y., Chen L., Zhu H., Hu C., Kang P., Liu L., Zhu H. (2020). Development and Validation of the HNC-LL Score for Predicting the Severity of Coronavirus Disease 2019. eBioMedicine.

[14] Wang J., Zhang H., Qiao R., Ge Q., Zhang S., Zhao Z., Tian C., Ma Q., Shen N. (2020). Thrombo-Inflammatory Features Predicting Mortality in Patients with COVID-19: The FAD-85 Score. J. Int. Med. Res..

[15] Knight S.R., Ho A., Pius R., Buchan I., Carson G., Drake T.M., Dunning J., Fairfield C.J., Gamble C., Green C.A. (2020). Risk Stratification of Patients Admitted to Hospital with COVID-19 Using the ISARIC WHO Clinical Characterisation Protocol: Development and Validation of the 4C Mortality Score. BMJ.

[16] Soto-Mota A., Marfil-Garza B.A., Martínez Rodríguez E., Barreto Rodríguez J.O., López Romo A.E., Alberti Minutti P., Alejandre Loya J.V., Pérez Talavera F.E., Ávila Cervera F.J., Velazquez Burciaga A. (2020). The Low-harm Score for Predicting Mortality in Patients Diagnosed with COVID-19: A Multicentric Validation Study. J. Am. Coll. Emerg. Physicians Open.

[17] Foieni F., Sala G., Mognarelli J.G., Suigo G., Zampini D., Pistoia M., Ciola M., Ciampani T., Ultori C., Ghiringhelli P. (2020). Derivation and Validation of the Clinical Prediction Model for COVID-19. Intern. Emerg. Med..

[18] Abdulaal A., Patel A., Charani E., Denny S., Alqahtani S.A., Davies G.W., Mughal N., Moore L.S.P. (2020). Comparison of Deep Learning with Regression Analysis in Creating Predictive Models for SARS-CoV-2 Outcomes. BMC Med. Inform. Decis. Mak..

[19] An C., Lim H., Kim D.-W., Chang J.H., Choi Y.J., Kim S.W. (2020). Machine Learning Prediction for Mortality of Patients Diagnosed with COVID-19: A Nationwide Korean Cohort Study. Sci. Rep..

[20] Chen H., Chen R., Yang H., Wang J., Hou Y., Hu W., Yu J., Li H. (2021). Development and Validation of a Nomogram Using on Admission Routine Laboratory Parameters to Predict In-hospital Survival of Patients with COVID-19. J. Med. Virol..

[21] Chen R., Liang W., Jiang M., Guan W., Zhan C., Wang T., Tang C., Sang L., Liu J., Ni Z. (2020). Risk Factors of Fatal Outcome in Hospitalized Subjects with Coronavirus Disease 2019 from a Nationwide Analysis in China. Chest.

[22] Cheng A., Hu L., Wang Y., Huang L., Zhao L., Zhang C., Liu X., Xu R., Liu F., Li J. (2020). Diagnostic Performance of Initial Blood Urea Nitrogen Combined with D-Dimer Levels for Predicting in-Hospital Mortality in COVID-19 Patients. Int. J. Antimicrob. Agents.

[23] Clift A.K., Coupland C.A.C., Keogh R.H., Diaz-Ordaz K., Williamson E., Harrison E.M., Hayward A., Hemingway H., Horby P., Mehta N. (2020). Living Risk Prediction Algorithm (QCOVID) for Risk of Hospital Admission and Mortality from Coronavirus 19 in Adults: National Derivation and Validation Cohort Study. BMJ.

[24] Gao Y., Cai G.-Y., Fang W., Li H.-Y., Wang S.-Y., Chen L., Yu Y., Liu D., Xu S., Cui P.-F. (2020). Machine Learning Based Early Warning System Enables Accurate Mortality Risk Prediction for COVID-19. Nat. Commun..

[25] Kamran S.M., Mirza Z.-H., Moeed H.A., Naseem A., Hussain M., Fazal I., Saeed F., Alamgir W., Saleem S., Riaz S. (2020). CALL Score and RAS Score as Predictive Models for Coronavirus Disease 2019. Cureus.

[26] Liang W., Liang H., Ou L., Chen B., Chen A., Li C., Li Y., Guan W., Sang L., Lu J. (2020). Development and Validation of a Clinical Risk Score to Predict the Occurrence of Critical Illness in Hospitalized Patients with COVID-19. JAMA Intern. Med..

[27] Liu J., Liu Z., Jiang W., Wang J., Zhu M., Song J., Wang X., Su Y., Xiang G., Ye M. (2021). Clinical Predictors of COVID-19 Disease Progression and Death: Analysis of 214 Hospitalised Patients from Wuhan, China. Clin. Respir. J..

[28] Lorente L., Martín M.M., Argueso M., Solé-Violán J., Perez A., Marcos y Ramos J.A., Ramos-Gómez L., López S., Franco A., González-Rivero A.F. (2020). Association between Red Blood Cell Distribution Width and Mortality of COVID-19 Patients. Anaesth. Crit. Care Pain Med..

[29] Ma X., Li A., Jiao M., Shi Q., An X., Feng Y., Xing L., Liang H., Chen J., Li H. (2020). Characteristic of 523 COVID-19 in Henan Province and a Death Prediction Model. Front. Public Health.

[30] Ma X., Ng M., Xu S., Xu Z., Qiu H., Liu Y., Lyu J., You J., Zhao P., Wang S. (2020). Development and Validation of Prognosis Model of Mortality Risk in Patients with COVID-19. Epidemiol. Infect..

[31] Ma X., Wang H., Huang J., Geng Y., Jiang S., Zhou Q., Chen X., Hu H., Li W., Zhou C. (2020). A Nomogramic Model Based on Clinical and Laboratory Parameters at Admission for Predicting the Survival of COVID-19 Patients. BMC Infect. Dis..

[32] Pan D., Cheng D., Cao Y., Hu C., Zou F., Yu W., Xu T. (2020). A Predicting Nomogram for Mortality in Patients with COVID-19. Front. Public Health.

[33] Pan P., Li Y., Xiao Y., Han B., Su L., Su M., Li Y., Zhang S., Jiang D., Chen X. (2020). Prognostic Assessment of COVID-19 in the Intensive Care Unit by Machine Learning Methods: Model Development and Validation. J. Med. Internet Res..

[34] Park J.G., Kang M.K., Lee Y.R., Song J.E., Kim N.Y., Kweon Y.O., Tak W.Y., Jang S.Y., Lee C., Kim B.S. (2020). Fibrosis-4 Index as a Predictor for Mortality in Hospitalised Patients with COVID-19: A Retrospective Multicentre Cohort Study. BMJ Open.

[35] Salto-Alejandre S., Roca-Oporto C., Martín-Gutiérrez G., Avilés M.D., Gómez-González C., Navarro-Amuedo M.D., Praena-Segovia J., Molina J., Paniagua-García M., García-Delgado H. (2021). A Quick Prediction Tool for Unfavourable Outcome in COVID-19 Inpatients: Development and Internal Validation. J. Infect..

[36] Santos-Lozano A., Calvo-Boyero F., López-Jiménez A., Cueto-Felgueroso C., Castillo-García A., Valenzuela P.L., Arenas J., Lucia A., Martín M.A., COVID-19 Hospital ’12 Octubre’ Clinical Biochemisty Study Group (2020). Can Routine Laboratory Variables Predict Survival in COVID-19? An Artificial Neural Network-Based Approach. Clin. Chem. Lab. Med..

[37] Turcotte J.J., Meisenberg B.R., MacDonald J.H., Menon N., Fowler M.B., West M., Rhule J., Qureshi S.S., MacDonald E.B. (2020). Risk Factors for Severe Illness in Hospitalized COVID-19 Patients at a Regional Hospital. PLoS ONE.

[38] Vaid A., Somani S., Russak A.J., De Freitas J.K., Chaudhry F.F., Paranjpe I., Johnson K.W., Lee S.J., Miotto R., Richter F. (2020). Machine Learning to Predict Mortality and Critical Events in a Cohort of Patients with COVID-19 in New York City: Model Development and Validation. J. Med. Internet Res..

[39] Varol Y., Hakoglu B., Kadri Cirak A., Polat G., Komurcuoglu B., Akkol B., Atasoy C., Bayramic E., Balci G., Ataman S. (2021). The Impact of Charlson Comorbidity Index on Mortality from SARS-CoV-2 Virus Infection and A Novel COVID-19 Mortality Index: CoLACD. Int. J. Clin. Pract..

[40] Wang B., Zhong F., Zhang H., An W., Liao M., Cao Y. (2020). Risk Factor Analysis and Nomogram Construction for Non-Survivors among Critical Patients with COVID-19. Jpn. J. Infect. Dis..

[41] Wang R., He M., Yin W., Liao X., Wang B., Jin X., Ma Y., Yue J., Bai L., Liu D. (2020). The Prognostic Nutritional Index Is Associated with Mortality of COVID-19 Patients in Wuhan, China. J. Clin. Lab. Anal..

[42] Wu G., Zhou S., Wang Y., Lv W., Wang S., Wang T., Li X. (2020). A Prediction Model of Outcome of SARS-CoV-2 Pneumonia Based on Laboratory Findings. Sci. Rep..

[43] Yadaw A.S., Li Y., Bose S., Iyengar R., Bunyavanich S., Pandey G. (2020). Clinical Features of COVID-19 Mortality: Development and Validation of a Clinical Prediction Model. Lancet Digit. Health.

[44] Yan L., Zhang H.-T., Goncalves J., Xiao Y., Wang M., Guo Y., Sun C., Tang X., Jing L., Zhang M. (2020). An Interpretable Mortality Prediction Model for COVID-19 Patients. Nat. Mach. Intell..

[45] Zhang S., Guo M., Duan L., Wu F., Hu G., Wang Z., Huang Q., Liao T., Xu J., Ma Y. (2020). Development and Validation of a Risk Factor-Based System to Predict Short-Term Survival in Adult Hospitalized Patients with COVID-19: A Multicenter, Retrospective, Cohort Study. Crit. Care.

[46] Zhao Z., Chen A., Hou W., Graham J.M., Li H., Richman P.S., Thode H.C., Singer A.J., Duong T.Q. (2020). Prediction Model and Risk Scores of ICU Admission and Mortality in COVID-19. PLoS ONE.

[47] Zinellu A., Arru F., De Vito A., Sassu A., Valdes G., Scano V., Zinellu E., Perra R., Madeddu G., Carru C. (2021). The De Ritis Ratio as Prognostic Biomarker of In-hospital Mortality in COVID-19 Patients. Eur. J. Clin. Investig..

[48] Hu C., Liu Z., Jiang Y., Shi O., Zhang X., Xu K., Suo C., Wang Q., Song Y., Yu K. (2020). Early Prediction of Mortality Risk among Patients with Severe COVID-19, Using Machine Learning. Int. J. Epidemiol..

[49] Bello-Chavolla O.Y., Bahena-López J.P., Antonio-Villa N.E., Vargas-Vázquez A., González-Díaz A., Márquez-Salinas A., Fermín-Martínez C.A., Naveja J.J., Aguilar-Salinas C.A. (2020). Predicting Mortality Due to SARS-CoV-2: A Mechanistic Score Relating Obesity and Diabetes to COVID-19 Outcomes in Mexico. J. Clin. Endocrinol. Metab..

[50] Torres-Macho J., Ryan P., Valencia J., Pérez-Butragueño M., Jiménez E., Fontán-Vela M., Izquierdo-García E., Fernandez-Jimenez I., Álvaro-Alonso E., Lazaro A. (2020). The PANDEMYC Score. An Easily Applicable and Interpretable Model for Predicting Mortality Associated with COVID-19. J. Clin. Med..

[51] Liu Q., Wang Y., Zhao X., Wang L., Liu F., Wang T., Ye D., Lv Y. (2021). Diagnostic Performance of a Blood Urea Nitrogen to Creatinine Ratio-Based Nomogram for Predicting In-Hospital Mortality in COVID-19 Patients. Risk Manag. Healthc. Policy.

[52] Magro B., Zuccaro V., Novelli L., Zileri L., Celsa C., Raimondi F., Gori M., Cammà G., Battaglia S., Genova V.G. (2021). Predicting In-Hospital Mortality from Coronavirus Disease 2019: A Simple Validated App for Clinical Use. PLoS ONE.

[53] Niu Y., Zhan Z., Li J., Shui W., Wang C., Xing Y., Zhang C. (2022). Development of a Predictive Model for Mortality in Hospitalized Patients with COVID-19. Disaster Med. Public Health Prep..

[54] Ding Z., Li G., Chen L., Shu C., Song J., Wang W., Wang Y., Chen Q., Jin G., Liu T. (2020). Association of Liver Abnormalities with In-Hospital Mortality in Patients with COVID-19. J. Hepatol..

[55] Zhang B., Liu Q., Zhang X., Liu S., Chen W., You J., Chen Q., Li M., Chen Z., Chen L. (2020). Clinical Utility of a Nomogram for Predicting 30-Days Poor Outcome in Hospitalized Patients with COVID-19: Multicenter External Validation and Decision Curve Analysis. Front. Med..

[56] Acar H.C., Can G., Karaali R., Börekçi Ş., Balkan İ.İ., Gemicioğlu B., Konukoğlu D., Erginöz E., Erdoğan M.S., Tabak F. (2021). An Easy-to-Use Nomogram for Predicting in-Hospital Mortality Risk in COVID-19: A Retrospective Cohort Study in a University Hospital. BMC Infect. Dis..

[57] Bellan M., Azzolina D., Hayden E., Gaidano G., Pirisi M., Acquaviva A., Aimaretti G., Aluffi Valletti P., Angilletta R., Arioli R. (2021). Simple Parameters from Complete Blood Count Predict In-Hospital Mortality in COVID-19. Dis. Markers.

[58] Besutti G., Ottone M., Fasano T., Pattacini P., Iotti V., Spaggiari L., Bonacini R., Nitrosi A., Bonelli E., Canovi S. (2021). The Value of Computed Tomography in Assessing the Risk of Death in COVID-19 Patients Presenting to the Emergency Room. Eur. Radiol..

[59] Chen B., Gu H.-Q., Liu Y., Zhang G., Yang H., Hu H., Lu C., Li Y., Wang L., Liu Y. (2021). A Model to Predict the Risk of Mortality in Severely Ill COVID-19 Patients. Comput. Struct. Biotechnol. J..

[60] Chowdhury M.E.H., Rahman T., Khandakar A., Al-Madeed S., Zughaier S.M., Doi S.A.R., Hassen H., Islam M.T. (2021). An Early Warning Tool for Predicting Mortality Risk of COVID-19 Patients Using Machine Learning. Cogn. Comput..

[61] Dong Y.-M., Sun J., Li Y.-X., Chen Q., Liu Q.-Q., Sun Z., Pang R., Chen F., Xu B.-Y., Manyande A. (2021). Development and Validation of a Nomogram for Assessing Survival in Patients with COVID-19 Pneumonia. Clin. Infect. Dis..

[62] Ebell M.H., Cai X., Lennon R., Tarn D.M., Mainous A.G., Zgierska A.E., Barrett B., Tuan W.-J., Maloy K., Goyal M. (2021). Development and Validation of the COVID-NoLab and COVID-SimpleLab Risk Scores for Prognosis in 6 US Health Systems. J. Am. Board Fam. Med..

[63] Guan X., Zhang B., Fu M., Li M., Yuan X., Zhu Y., Peng J., Guo H., Lu Y. (2021). Clinical and Inflammatory Features Based Machine Learning Model for Fatal Risk Prediction of Hospitalized COVID-19 Patients: Results from a Retrospective Cohort Study. Ann. Med..

[64] Harmouch F., Shah K., Hippen J.T., Kumar A., Goel H. (2021). Is It All in the Heart? Myocardial Injury as Major Predictor of Mortality among Hospitalized COVID-19 Patients. J. Med. Virol..

[65] He J., Song C., Liu E., Liu X., Wu H., Lin H., Liu Y., Li Q., Xu Z., Ren X. (2021). Establishment of Routine Clinical Indicators-Based Nomograms for Predicting the Mortality in Patients with COVID-19. Front. Med..

[66] Jiang M., Li C., Zheng L., Lv W., He Z., Cui X., Dietrich C.F. (2021). A Biomarker-Based Age, Biomarkers, Clinical History, Sex (ABCS)-Mortality Risk Score for Patients with Coronavirus Disease 2019. Ann. Transl. Med..

[67] Ke Z., Li L., Wang L., Liu H., Lu X., Zeng F., Zha Y. (2022). Radiomics Analysis Enables Fatal Outcome Prediction for Hospitalized Patients with Coronavirus Disease 2019 (COVID-19). Acta Radiol..

[68] Kim D.H., Park H.C., Cho A., Kim J., Yun K., Kim J., Lee Y.-K. (2021). Age-Adjusted Charlson Comorbidity Index Score Is the Best Predictor for Severe Clinical Outcome in the Hospitalized Patients with COVID-19 Infection. Medicine.

[69] Li J., Yang L., Zeng Q., Li Q., Yang Z., Han L., Huang X., Chen E. (2021). Determinants of Mortality of Patients with COVID-19 in Wuhan, China: A Case-Control Study. Ann. Palliat. Med..

[70] Li L., Fang X., Cheng L., Wang P., Li S., Yu H., Zhang Y., Jiang N., Zeng T., Hou C. (2021). Development and Validation of a Prognostic Nomogram for Predicting In-Hospital Mortality of COVID-19: A Multicenter Retrospective Cohort Study of 4086 Cases in China. Aging.

[71] Moon H.J., Kim K., Kang E.K., Yang H.-J., Lee E. (2021). Prediction of COVID-19-Related Mortality and 30-Day and 60-Day Survival Probabilities Using a Nomogram. J. Korean Med. Sci..

[72] Ottenhoff M.C., Ramos L.A., Potters W., Janssen M.L.F., Hubers D., Hu S., Fridgeirsson E.A., Piña-Fuentes D., Thomas R., van der Horst I.C.C. (2021). Predicting Mortality of Individual Patients with COVID-19: A Multicentre Dutch Cohort. BMJ Open.

[73] Pfeifer N., Zaboli A., Ciccariello L., Bernhart O., Troi C., Fanni Canelles M., Ammari C., Fioretti A., Turcato G. (2022). Nomogramm zur Risikostratifizierung von COVID-19-Patienten mit interstitieller Pneumonie in der Notaufnahme: Eine retrospektive multizentrische Studie. Med. Klin. Intensivmed. Notfallmed..

[74] Rahman T., Al-Ishaq F.A., Al-Mohannadi F.S., Mubarak R.S., Al-Hitmi M.H., Islam K.R., Khandakar A., Hssain A.A., Al-Madeed S., Zughaier S.M. (2021). Mortality Prediction Utilizing Blood Biomarkers to Predict the Severity of COVID-19 Using Machine Learning Technique. Diagnostics.

[75] Rahman T., Khandakar A., Hoque M.E., Ibtehaz N., Kashem S.B., Masud R., Shampa L., Hasan M.M., Islam M.T., Al-Maadeed S. (2021). Development and Validation of an Early Scoring System for Prediction of Disease Severity in COVID-19 Using Complete Blood Count Parameters. IEEE Access.

[76] Sánchez-Montañés M., Rodríguez-Belenguer P., Serrano-López A.J., Soria-Olivas E., Alakhdar-Mohmara Y. (2020). Machine Learning for Mortality Analysis in Patients with COVID-19. Int. J. Environ. Res. Public Health.

[77] Wang H., Ai H., Fu Y., Li Q., Cui R., Ma X., Ma Y., Wang Z., Liu T., Long Y. (2021). Development of an Early Warning Model for Predicting the Death Risk of Coronavirus Disease 2019 Based on Data Immediately Available on Admission. Front. Med..

[78] Wang K., Zuo P., Liu Y., Zhang M., Zhao X., Xie S., Zhang H., Chen X., Liu C. (2020). Clinical and Laboratory Predictors of In-Hospital Mortality in Patients with Coronavirus Disease-2019: A Cohort Study in Wuhan, China. Clin. Infect. Dis..

[79] Xiao F., Sun R., Sun W., Xu D., Lan L., Li H., Liu H., Xu H. (2021). Radiomics Analysis of Chest CT to Predict the Overall Survival for the Severe Patients of COVID-19 Pneumonia. Phys. Med. Biol..

[80] Yu J., Nie L., Wu D., Chen J., Yang Z., Zhang L., Li D., Zhou X. (2021). Prognostic Value of a Clinical Biochemistry-Based Nomogram for Coronavirus Disease 2019. Front. Med..

[81] Xiao M., Hou M., Liu X., Li Z., Zhao Q. (2020). Clinical characteristics of 71 patients with coronavirus disease 2019. J. Cent. South Univ. Med. Sci..

[82] Magro F., Gionchetti P., Eliakim R., Ardizzone S., Armuzzi A., Barreiro-de Acosta M., Burisch J., Gecse K.B., Hart A.L., Hindryckx P. (2017). Third European Evidence-Based Consensus on Diagnosis and Management of Ulcerative Colitis. Part 1: Definitions, Diagnosis, Extra-Intestinal Manifestations, Pregnancy, Cancer Surveillance, Surgery, and Ileo-Anal Pouch Disorders. J. Crohns Colitis.

[83] Dorjee K., Kim H., Bonomo E., Dolma R. (2020). Prevalence and Predictors of Death and Severe Disease in Patients Hospitalized Due to COVID-19: A Comprehensive Systematic Review and Meta-Analysis of 77 Studies and 38,000 Patients. PLoS ONE.

[84] Zhao Q., Meng M., Kumar R., Wu Y., Huang J., Lian N., Deng Y., Lin S. (2020). The Impact of COPD and Smoking History on the Severity of COVID-19: A Systemic Review and Meta-Analysis. J. Med. Virol..

[85] Bhattacharyya A., Seth A., Srivast N., Imeokparia M., Rai S. (2021). Coronavirus (COVID-19): A Systematic Review and Meta-Analysis to Evaluate the Significance of Demographics and Comorbidities. Res. Sq..

[86] Ho J.S.Y., Fernando D.I., Chan M.Y., Sia C.H. (2020). Obesity in COVID-19: A Systematic Review and Meta-Analysis. Ann. Acad. Med. Singap..

[87] Zhao B.-C., Liu W.-F., Lei S.-H., Zhou B.-W., Yang X., Huang T.-Y., Deng Q.-W., Xu M., Li C., Liu K.-X. (2020). Prevalence and Prognostic Value of Elevated Troponins in Patients Hospitalised for Coronavirus Disease 2019: A Systematic Review and Meta-Analysis. J. Intensive Care.

[88] Del Zompo F., De Siena M., Ianiro G., Gasbarrini A., Pompili M., Ponziani F.R. (2020). Prevalence of Liver Injury and Correlation with Clinical Outcomes in Patients with COVID-19: Systematic Review with Meta-Analysis. Eur. Rev. Med. Pharmacol. Sci..

[89] Silver S.A., Beaubien-Souligny W., Shah P.S., Harel S., Blum D., Kishibe T., Meraz-Munoz A., Wald R., Harel Z. (2020). The Prevalence of Acute Kidney Injury in Patients Hospitalized with COVID-19 Infection: A Systematic Review and Meta-Analysis. Kidney Med..

[90] Ruan Q., Yang K., Wang W., Jiang L., Song J. (2020). Clinical Predictors of Mortality Due to COVID-19 Based on an Analysis of Data of 150 Patients from Wuhan, China. Intensive Care Med..

[91] Steyerberg E. (2009). Clinical Prediction Models: A Practical Approach to Development, Validation, and Updating.

[92] Zhang H., Shi T., Wu X., Zhang X., Wang K., Bean D., Dobson R., Teo J.T., Sun J., Zhao P. (2020). Risk Prediction for Poor Outcome and Death in Hospital In-Patients with COVID-19: Derivation in Wuhan, China and External Validation in London, UK. medRxiv.

