# Supplementary material for: Predicting In-Hospital Mortality in Severe COVID-19: A Systematic Review and External Validation of Clinical Prediction Rules
Source: Biomedicines. 2022 Sep 27;10(10):2414. doi: 10.3390/biomedicines10102414 (PMC9599062; doi:10.3390/biomedicines10102414)
Supplement: Supplementary file 1 [file biomedicines-10-02414-s001.zip › Supplementary Table S1.pdf]

Supplementary Table S1. Search strategies carried out in four bibliographic databases for documents that report on clinical prediction models for hospital mortality caused by COVID-19

Database	Search Strategy	Items
PubMed	((Clinical Decision Rules OR nomograms)) AND (covid* OR sars*) AND mortality	106
Scopus	#1: Clinical Decision Rules OR Nomograms	85279
	#2: Mortality	4022668
	#3: Covid* OR SARS*	711600
	#1 AND #2 AND *3:* ("Clinical Decision Rules" OR Nomograms) AND (Mortality) AND (Covid* OR SARS*)	375
Web Of Science	#1: Clinical Decision Rules OR Nomograms	26299
	#2: Mortality	1054626
	#3: Covid* OR SARS*	217115
	#1 AND #2 AND *3:* ("Clinical Decision Rules" OR Nomograms) AND (Mortality) AND (Covid* OR SARS*)	79
EMBASE	('clinical decision rules'/exp OR 'clinical decision rules' OR (('clinical'/exp OR clinical) AND ('decision'/exp OR decision) AND rules) OR 'nomograms'/exp OR nomograms) AND (covid* OR sars*) AND ('mortality'/exp OR mortality)	53
