# Supplementary material for: Predicting In-Hospital Mortality in Severe COVID-19: A Systematic Review and External Validation of Clinical Prediction Rules
Source: Biomedicines. 2022 Sep 27;10(10):2414. doi: 10.3390/biomedicines10102414 (PMC9599062; doi:10.3390/biomedicines10102414)
Supplement: Supplementary file 1 [file biomedicines-10-02414-s001.zip › Supplementary Table S2.pdf]

Supplementary Table S2. Overview of prediction models for mortality risk from Covid-19 identified in a systematic review of the literature, and performance of each model in derivation cohorts. Formulas to calculate mortality risk to be applied to any population were provided or extracted from original documents.

Author, Year[reference]	Country	Study period (2020)	N	Methods used to derivate nomograms	Predictors in final model	AUROC (95% confidence interval)	Cut-off value	Sensitivity (%)	Specificity (%)	Positive predictive value (%)	Negative predictive value (%)
Abdulaal A et al. 2020[18]	United Kingdom	1 Mar – 24 Apr	**398** (**D:** 318 **V:** 80)	Cox regression model	Age; Sex; Obesity; ischaemic heart disease; cardiac failure; chronic liver disease; chronic kidney disease; cerebrovascular event; cough; dyspnoea; abdominal pain; confusion; collapse.	0.869 (0.857 – 0.882)	50%	50	96.6	84.6	83.6
				ANN	Same variables + hypertension; diabetes, NOD; chronic respiratory disease; fever; diarrhoea or vomiting; myalgia and olfactory change.	0.926 (0.911 – 0.941)		64.7	96.8	84.6	91.4
	**h(t) = h0(t) × exp(0.05 × age- + (−0.78 × sex) + 1.48 × obesity + (0.93 × ischaemic heart disease) + 0.91 × cardiac failure + (1.36 × chronic liver disease) + (1.03 × chronic kidney disease) + (0.78 × cerebrovascular event) + 1.01 × cough + 1.54 × dyspnoea + (−1.45 × abdominal pain) + 1.81 × confusion + (1.35 × collapse)**										
Acar HC et al. 2021[56]	Turkey	16 March – 18 June	**709**	Multivariate logistic regression	Age; Comorbidity; Dyspnoea; SpO_2; haematocrit; CRP; AST and ferritin	0.922 (0.89 - 0.96)	<17 points (Low Risk (<10%)) >38 points (High Risk (>90%))	NA	NA	NA	NA
	**Total Score: Age [55-64 (4) / 65-74 (8) / >75 (12)] + Comorbidity (3) + Dyspnoea (2) + SpO2 (%) [89-94 (3) / <88 (9)] + Haematocrit (%) [<35% (3)] + CRP (mg/L) [>50 - <100 (2) / ≥ 100 (6)] + AST (IU/L) [>40 - <80 (2) / ≥ 80 (7)] + Ferritin (ng/mL) [>400 - <1000 (2) / ≥ 1000 (5)]**										
An C et al. 2020[19]	Korea	23 Jan - 2 April	**10237**	LASSO	age >80; acarbose; age >70; metformin; cancer; age >60; nursing home infection; metropolitan; mental illness; cluster infection; urban; lung disease or asthma, fenofibrate; male; disability >moderate; symptomatic; ACE inhibitor; beta blocker; age >40; seniors living alone; loop diuretics; cerebrovascular disease; income (50-75%); DDP-4 inhibitor; cardiovascular disease; sulfonylurea; chronic renal disease; hypertension; Income (medicaid); age <40; aspirin; DM; personal contact; statin; income (25-50%); chronic liver disease; calcium channel blocker; income (>75%); disability mild; NSAID; hyperlipidaemia and AR blocker.	0.963 (0.946 - 0.979)	ND	90.7	91.4	24.6	99.7
				Linear SVM		0.962 (0.945 - 0.979)		92.0	91.8	25.7	99.7
				RBF-SVM		0.958 (0.945 - 0.971)		42.7	97.8	37.6	98.2
				RF		0.958 (0.936 - 0.981)		32.0	98.8	44.4	97.9
				KNN		0.897 (0.856 -0.937)		81.3	89.5	19.3	99.4
Bellan M et al. 2021[57]	Italy	1 Mar – 28 April	**664** **V**:bootstrap	Multivariate regression	Age (<74); Male gender; Platelets (<166000); NLR (>4.68) and RDW (>13.7%)	No global (individually stated)	ND	NA	NA	NA	NA

Author, Year[reference]	Country	Study period (2020)	N	Methods used to derivate nomograms	Predictors in final model	AUROC (95% confidence interval)	Cut-off value	Sensitivity (%)	Specificity (%)	Positive predictive value (%)	Negative predictive value (%)
Bello-Chavolla OY et al. 2020[49]	Mexico	To 18 May	**177133**	Cox proportional risk model	Age; diabetes; obesity; pneumonia; CKD; COPD and immunosuppression.	0.823 (C-Statistic)	Low Risk ≤ 0 points Mild Risk 1- 3 points Moderate Risk 4- 6 points High Risk 7 – 9 points	NA	NA	NA	NA
	**Total Score: Immunosuppression x1 + COPD x1 + CKD x3 + Pneumonia x7 + Obesity x1 + Age<40 x(-6) + Age>=65 x 3 + Diabetes x1 + Diabetes*Age<40 x 5**										
Besutti G et al. 2021[58]	Italia	27 Feb – 23 Mar	**886** **V**:bootstrap	Cox regression	Age; Gender; HDL cholesterol; Dementia; Heart failure; Vascular diseases: Days from symptoms onset; Neutrophils; LDH; SO2 and CT extension	**D:** 0.947 (0.930 – 0.964) **V:** 0.936 (0.912 – 0.953)	ND	NA	NA	NA	NA
Chen B et al. 2021[59]	China	16 Feb – 14 Apr	**1002** (**D:** 566 **V:** 436)	Multivariate logistic regression and LASSO	Age; Chronic lung disease; NLR; Creatinine; D-dimer; CRP and Total bilirubin	**D:** 0.912 (0.88 – 0.94) **V:** 0.922 (0.89 – 0.95)	ND	NA	NA	NA	NA
Chen H et al. 2020[20]	China	9 Feb - 30 Ma	**855**	Cox regression and LASSO	Neutrophils; CRP; IL-6; prothrombin time; myoglobin and D-dimer	0.912 (C-Statistic)	Very High Risk ≥ 10 points	NA	NA	NA	NA
Chen R et al. 2020[21]	China	Up to 31 Jan	**1590**	Cox regression	Age; coronary heart disease; cerebrovascular disease; dyspnoea; procalcitonin and AST	0.91 (0.85 - 0.97) (C-Statistic)	NA	NA	NA	NA	NA
Cheng A et al. 2020[22]	China	8 Feb - 11	**305**	Cox regression	BUN and D-dimer	0.94 (0.90-0.97)	BUN >=4,6 & D-dimer >=0,845	85	91	NA	NA
	**Total Score: BUN + D-dimer**										
Chowdhury MEH et al. 2020[60]	China	10 Jan – 16 Feb	**375** **V**:bootstrap	Multivariate logistic regression and Machine Learning	Age; hsCRP; Lymphocytes; Neutrophils and LDH	**D:** 0.961 **V:** 0.991	<10.4 (Low Risk (<5%)<br>10.4 – 12.65 (Moderate Risk (5 -50%)<br>> 12.65 (High Risk (>50%)	59.7<br><br>89.2	100<br><br>90.2	100<br><br>91.2	68.7<br><br>88.1
	**Total Score: -3.662636 + 0.0735038*Age (years) + 0.0110451*hsCRP (mg/L) – 0.1624422*Lymphocytes (%) – 0.0327053*Neutrophils (%) + 0.0070514*LDH (U/L)**										

Author, Year[reference]	Country	Study period (2020)	N	Methods used to derivate nomograms	Predictors in final model	AUROC (95% confidence interval)	Cut-off value	Sensitivity (%)	Specificity (%)	Positive predictive value (%)	Negative predictive value (%)
Clift AK et al. 2020[23]	United Kingdom	24 Jan - 30 Apr	**10776**	LASSO	Ethnic group; nursing home or homeless; disability; chronic kidney disease; chemotherapy; blood cancer; bone marrow or stem cell transplant in past 6 months; respiratory tract cancer; radiotherapy in past 6 months; solid organ transplant (excluding kidney and bone marrow); immunosuppressant medication from GP 4+ scripts in past 6 months; leukotriene or long acting β agonist 4+ scripts in past 6 months; oral steroids 4+ scripts in past 6 months; sickle cell disease or severe immunodeficiency; diabetes; COPD; asthma; rare lung conditions; pulmonary hypertension or pulmonary fibrosis; coronary heart disease; stroke; atrial fibrillation; congestive cardiac failure; thromboembolism; peripheral vascular disease; congenital heart disease; dementia; Parkinson's disease; epilepsy; motor neurone disease, multiple sclerosis, myasthenia gravis, or Huntington's; cerebral palsy; severe mental illness; osteoporotic fracture; rheumatoid arthritis or SLE; cirrhosis of liver	C- Statistic<br><br>Men: 0.928 (0.919 - 0.938)<br><br>Women: 0.933 (0.923 - 0.944)	NA	NA	NA	NA	NA
Ding Z et al. 2021[54]	China	18 Jan- 25 Apr	**2073**	Cox regression	Age; severe pneumonia; lymphocyte count; platelets; CRP; D-dimer; creatinine; cTnl; AST and D-bilirubine.	0.887 (0.844 - 0.929) C- Statistic	NA	NA	NA	NA	NA

Author, Year[reference]	Country	Study period (2020)	N	Methods used to derivate nomograms	Predictors in final model	AUROC (95% confidence interval)	Cut-off value	Sensitivity (%)	Specificity (%)	Positive predictive value (%)	Negative predictive value (%)
Dong YM et al 2021[61]	China	Jan - Mar	**628** **D:** 390 **V:**238	Cox regression and LASSO	Hypertension; NLR; NT-proBNP	**D:** 0.922 (14day) / 0.919 (21day) **V:** 0.922 (14day) / 0.881 (21 day)	NA	NA	NA	NA	NA
Ebell MH et al 2021[62]	USA	1 Mar – 12 Jun	**1442** **NoLab:** 1342 (60% D / 40%V)	Multivariate logistic regression	**NoLab:** Age; Respiratory Rate and O_2 saturation	**D:** 0.771 **V:** 0.803	Low (0 – 1 points)	2.5	76.1	1.8	81.5
							Moderate (2 – 5)				
							High (≥ 6 points)	36.4	92.4	45.8	89.2
			**SimpleLab:** 741 (60% D / 40%V)		**SimpleLab:** Age; Respiratory Rate and O_2 saturation; CRP; Asthma; WBC and serum creatinine	**D:** 0.835 **V:** 0.833	Low (0 – 7 points)	-	-	-	-
							Moderate (8 – 11)				
							High (≥ 12 points)	85.3	66.3	31.4	96.2
	**Total NoLab Score: Age [50 to 65 years (3 points) > 65 years (5 points)] + Respiratory Rate [≥ 30 breaths/min (3 points)] + O_2 saturation [>93% (2 points)]**										
	**Total SimpleLab Score: [50 to 65 years (6 points) > 65 years (8 points)] + Respiratory Rate [≥ 30 breaths/min (5 points)] + O_2 saturation [>93% (4 points)] + CRP [>10 mg/dL (5 points)] + Asthma [Yes (4 points)] + WBC [>10 x 10^9/mL (3 points)] + serum creatinine [>2 mg/dL (4 points)]**										
Foieni F et al. 2020[17]	Italy	15 Mar - 30 Apr	**119** (**D:** 79 **V:** 40)	Multiple linear regression	Age; fever +5 days; hypertension; Patter US "Wet"; P/F ratio; Lactates; WBC and CRP	0,90 (0,801 - 0,982)	Group 1: 0.3087–0.9391; Group 2: 0.9392–1.5696; Group 3: 1.5697–2.2000; Group 4: 2.2001–2.8304	NA	NA	NA	NA
	**Total Score: -0,998 + (Age*0,014) + (Fever*0,213) + (Hypertension*0,194) + (US*0,731) + (P/F*0,002) + (Lactate*0,041) + (WBC*-0,022) + (CPR*0,019) https://health-key.it/**										
Gao Y et al. 2020[24]	China	27 Jan – 21 Mar	**2160**	MRPMC	Respiratory rate; BUN; D-dimer; age; platelets; Sp02; lymphocytes; albumin; sex; fever; number comorbidities; sputum; chronic kidney disease and consciousness	0.9621 (0.946 – 0 978)	60% (0.6)	57.3	98.3	85	93.2
				SVM		0.9594 (0.942 – 0.976)		60.7	97.8	81.8	93.7
				GBDT		0.9454 (0.925 – 0.966)		60.7	96.6	75	93.6
				Logistic Regression		0.9614 (0.946 – 0.977)		56.2	98.1	83.3	93.1
				KNN		0.9615 (0.946 – 0.977)		51.7	98.9	88.5	92.5

Author, Year[reference]	Country	Study period (2020)	N	Methods used to derivate nomograms	Predictors in final model	AUROC (95% confidence interval)	Cut-off value	Sensitivity (%)	Specificity (%)	Positive predictive value (%)	Negative predictive value (%)
Guan X et al. 2021[63]	China	27 Jan - 5 Apr	**1270**<br>**D:** 984<br>**V:**286	Cox regression, LASSO and XGBoost	Disease severity; Age; hsCRP; LDH; ferritin and LDH	**D:** 0.921<br>**V:** 0.891	NA	NA	NA	NA	NA
Harmouch F et al. 2020[64]	USA	1 Mar – 15 Apr	**560**	Multivariate logistic regression	Age and Troponin (≥0.05ng/ml)	0.793<br>(0.733 – 0.853)	NA	NA	NA	NA	NA
He J et al. 2021[65]	China	4 Feb – 31 Mar	**3611**<br>**D:** 2119<br>**V:** 1412	Multivariate logistic regression	**Nomo1:** Age; Dyspnea; Anorexia; WBC; NLR; PLT; AST; Albumin and CRP<br><br>**Nomo2:** Age; Dyspnea; NLR and CRP	**D:** 0.920<br>(0.882 – 0.957)<br>**V:** 0.92<br>(0.86 – 0.98)<br>**D:** 0.896<br>(0.855 – 0.936)<br>**V:** 0.89<br>(0.83 – 0.96)	NA	86.7<br><br>76.7	88.7<br><br>89.8	NA<br><br>NA	NA<br><br>NA
Hu C et al. 2020[48]	China	28 Jan - 11 Mar	**284**<br>**(D:** 184<br>**V:** 64)	Random Forest<br>Bagged FDA<br>Logistic Regression<br>PLS<br>Elastic net	Age; lymphocytes; D-dimer and CRP	0.922<br>0.899<br>0.895<br>0.885<br>0.884	50%	91.4<br>88.1<br>89.2<br>91.2<br>91.2	76<br>73<br>68.7<br>66.3<br>64.7	NA<br>NA<br>NA<br>NA<br>NA	NA<br>NA<br>NA<br>NA<br>NA
	**Total Score: -4,211 + 0,013xCRP + 0,059xAge + 0,112xDD - 1,984xLym**<br>https://phenomics.fudan.edu.cn/risk_scores/										
Ji D et al. 2020[12]	China	20 Jan - 22 Feb	**208**	Cox regression	Comorbidity; age; lymphocyte and LDH	0.91<br>(0.86 – 0.94)	6 points<br>9 points	95<br>45	78<br>97	50.7<br>78.3	98.5<br>11.9
	**Total Score: (Comorbidity x4) + (Age> 60 x3) + (Lymphocytes ≤ 1 x 109 x3) + (LDH >500 x3 ó LDH 250 - 500 x2)**										
Jian M et al. 2021[66]	China	**D:** 1 Jan – 10 Apr<br>**V:** 13 Jan – 25 Mar	**1905**<br>**D:** 1717<br>**V:** 188	Cox regression	Age; Gender; COPD; AST; hsCRP; hsTNI; WBC; Lymphocyte count; D-dimer and Procalcitonin	**D:** C- Statistic 0.888<br>(0.869 – 0.0.907)<br>**V:** C- Statistic 0.838<br>(0.777– 0.899)	Low Risk (<2%)<br>Moderate Risk (2 -9%)<br>High Risk (>9%)	NA	NA	NA	NA
	**Total Score: Age [10y (0 points) / 20y (11 points) / 30y (22 points) / 40y (33 points) / 50y (44 points) / 60y (56 points) / 70y (67 points) / 80y (78 points) / 90y (89 points) / 100y (100 points)] + Gender [Female (0 points) Male (20 points)] + COPD [No (0 points Yes (23 points)] + AST [≤ 40 U/L (0 points) >40 U/L (22 points)] + hsCRP [≤ 10 mg/L (0) >10 mg/L (50 POINTS)] + hsTNI [≤15.6 (0 points) >15.6 (52 points)] + WBC [4-10 10^9/L (0 points) <4 10^9/L (7 points) ≥ 10 10^9/L (40 points)] + Lymphocyte count [≥ 1 10^9/L (0 points) ≥0.8 to <1 10^9/L (1 point) ≥ 0.5 to <0.8 10^9/L (12 points) <0.5 10^9/L (43 points)] + D-dimer [≤ 0.5 μg/L (0 points) ≥0.5 to <1 μg/L (7 points) ≥ 1 μg/L (37 points)] + Procalcitonin [<0.05 ng/mL(0 points) ≥0.05 to <0.5 ng/mL (37 points) ≥0.5 to <2 ng/mL (54 points) ≥ 2 ng/mL (69 points)]**										

**Author, Year**[reference]	**Country**	**Study period (2020)**	**N**	**Methods used to derivate nomograms**	**Predictors in final model**	**AUROC (95% confidence interval)**	**Cut-off value**	**Sensitivity (%)**	**Specificity (%)**	**Positive predictive value (%)**	**Negative predictive value (%)**
Kamran SM et al. 2020[25]	Pakistan	10 Apr-31 Aug	**252**	Cox regression	Respiratory rate; resting oxygen saturation and alveolar-arterial oxygen gradient	0.850 (0.80 - 0.89)	9 points	80	84	55	95
	**Total Score: Respiratory rate (≤20 x1; 21-25 x2; 26-30 x3; >30x4) + Resting oxygen saturation (≥95 x1; 90-94 x2; <90 x3) + A-a gradient (normal x1; elevated x3)**										
Kim DH et al. 2021[68]	Korea	Up to 30 Apr	**5621**	Cox regression	Sex; BMI; CCIS; Dyspnea; Hemoglobin; Lymphocyte and Platelet	C- Statistic 0.933	NA	NA	NA	NA	NA
Ke Z et al. 2022[67]	China	Jan - Mar	**96**<br>**D:** 66<br>**V:** 30	Cox regression and LASSO	Age; Temperature; Oxygen saturation; initial oxygen mode; Lymphocyte count and Radscore	C- Statistic<br>**D:** 0.912 (0.867– 0.907)<br>**V:** 0.907 (0.849– 0.966)	NA	NA	NA	NA	NA
Knight SR et al. 2020[15]	United Kingdom	6 Feb - 20 May	**57824**<br>(**D:** 35463<br>**V:** 22361)	Machine learning	Age; sex; number comorbidities; respiratory rate; peripheral oxygen saturation; Glasgow scale; urea level and CPR	0.786 (0.781 – 0.790)	> 9 points<br>> 15 points	92.5<br>38	38.6<br>89.8	39.3<br>61.5	92.3<br>77.1
	**Total Score: Age (<50; 50 - 59; +2; 60 - 69; +4; 70- 79; +6; >=80; +7) + Sex (Female; Male +1) + Nº comorbidities (0; 1; +1; >=2; +2) + Respiratory Rate (breaths/min) (<20; 20 - 29; +1 ; >=30; +2) + peripheral oxygen saturation (>=92; <92; +2) + Glasglow scale (15; <15; +2) + Urea level (mmol/L) (<7; 7- 14; +1; >14; +3) + RCP (mg/L) (<50; 50 - 99; +1; >=100; +2)**<br>https://isaric4c.net/risk/v2/										
Li J et al. 2021[69]	China	8 Jan – 17 Mar	**326**	Multivariate logistic regression	Age; CVD; WBC; Platelet; AST; Cystatin C, CRP; CKMB and D-dimer	C- Statistic 0.903	NA	NA	NA	NA	NA
Li L et al. 2021[70]	China	**D**: 13 Feb – 5 Mar<br>**V**: 5 Mar – 10 Apr	**4086**<br>(**D**: 1780<br>**IV**: 1242<br>**EV**: 1064)*	Cox regression	Age; Severity at admission; Dyspnoea; Cardiovascular disease; Total bilirrubin; Blood glucose; Urea and LDH	C- Statistic<br>**D:** 0.97 (0.95 – 0.98)<br>**IV:** 0.96 (0.94 – 0.98)<br>**EV:** 0.92 (0.86 – 0.98)	NA	NA	NA	NA	NA
Liang W et al. 2020[26]	China	21 Nov -31 Jan	**1590**	Logistic regression and LASSO	Chest radiographic abnormality; age; haemoptysis; dyspnoea; unconsciousness, number comorbidities; cancer history; NLR; LDH and direct bilirubin.	0.88 (0.85 - 0.91)	NA	NA	NA	NA	NA
	**Total Score: (X-ray abnormality *27.1) + (Age *0.614) + (Haemoptysis *33.6) + (Dyspnoea * 14.1) + (unconsciousness * 34.5) + (number comorbidities *10.4) + (cancer history*31.2) + (NLR*1.25) + (LDH *0.0534) + (direct bilirubin*3.06)**<br>http://118.126.104.170/										

Author, Year[reference]	Country	Study period (2020)	N	Methods used to derivate nomograms	Predictors in final model	AUROC (95% confidence interval)	Cut-off value	Sensitivity (%)	Specificity (%)	Positive predictive value (%)	Negative predictive value (%)
Liu J et al. 2020[27]	China	30 Jan - 20 Feb	**214**	Cox regression	Age; lymphocytes; LDH and procalcitonin	0.78 (0.73 - 0.83)	NA	NA	NA	NA	NA
Liu Q et al. 2021[51]	China	10 Jan – 27 Feb	**416** (**D:** 337 **V:** 79)	Logistic regression and LASSO	Age; gender and BUN; creatinine ratio	0.838 (0.795 – 0.880) (C- Statistic)	59.8 points	74.5	81	NA	NA
Lorente L et al. 2020[28]	Spain	-	**143**	-	RDW	0.71 (0.63 – 0.78)	13%	88	45	25	95
Ma X et al. 2020[29]	China	22 Jan - 20 Feb	**523**	Random forest	Pulse oxygen saturation <90%; age; creatinine; creatine kinase; D-dimer; neutrophil percentage; LDH; Leucocyte count; albumin; time form illness onset to hospital admission; glutamic-oxalacetic transaminase; neutrophil count; lymphocyte percentage; respiratory rate >24 breaths per min; prothrombin time; BUN; platelet; direct bilirubin; CRP; incubation period; eosinophil percentage; temperature; chronic respiratory disease; COPD; chest tightness; diabetes; coronary heart disease; chest CT; cardiovascular disease; hypertension; dyspnoea; cluster and Expectoration	0.9852 (0.961 – 1)	0.441	85	98.7	NA	NA
Ma X et al. 2020[30]	China	15 Jan - 15 Mar	**292**	Multivariate logistic regression	LDH; CRP and Age	0.952	NA	NA	NA	NA	NA
	**Total Score: −10.5772 + 0.0076 LDH + 0.00175 CRP + 0.0857 Age**										
Ma X et al. 2020[31]	China	12 Jan - 20 Mar	**262**	Logistic regression	Age; chronic heart disease; lymphocytes; platelets; CRP; LDH and D-Dimer	0.948 (0.923 – 0.973)	NA	NA	NA	NA	NA

Author, Year[reference]	Country	Study period (2020)	N	Methods used to derivate nomograms	Predictors in final model	AUROC (95% confidence interval)	Cut-off value	Sensitivity (%)	Specificity (%)	Positive predictive value (%)	Negative predictive value (%)
Magro B et al. 2021[52]	Italy	22 Feb - 7 Apr	**2191** (**D:** 1810 **V:** 381)	Cox regression	Age; gender; LDH; ischemic heart disease; liver disease; diabetes and days elapsed from first symptoms before hospitalization	0.822 (0.722 – 0.922)	NA	NA	NA	NA	NA
	**https://sites.google.com/community.unipa.it/covid-19riskpredictions/c19-rp**										
Moon HJ et al. 2021[71]	South Korea	1 Feb – 30 Apr	**5626**	Cox regression and Multivariate logistic regression	Age; Gender; Fever; Dyspnoea; DM; Cancer and Dementia	Overall mortality 0.959 (0.945 – 0.933)	NA	NA	NA	NA	NA
	**Multivariate logistic regression total score: -7.05 + 2.81*Age (years) + 1.08*Male gender + 0.27*Fever + 2.14*Dyspnoea + 1.17*DM + 1.38*Cancer +2.12*Dementia**<br>**Cox regression total score: 2.58*Age (years) + 0.84*Male gender + 0.67*Fever + 1.58*Dyspnoea + 0.69*DM + 0.82*Cancer +1.49*Dementia**										
Niu Y et al. 2021[53]	China	23 Jan - 5 Mar	**662** (**D:** 150 **V:** 512)	Multivariate logistic regression	Diabetes; LDH and SOFA Score	0.970 (0.947 – 0.992)	-	90.3	90.8	NA	NA
	**Total Score: -5.594 +(1.124 x SOFA) + (2.578 x LDH ([LDH≤245] = 0, [LDH>245] = 1) + (2.349 x Diabetes (without diabetes = 0, with diabetes = 1))**										
Ottenhoff MC et al. 2021[72]	Dutch	27 Feb – 8 Jun	**2273**	Multivariate logistic regression	Age; Number of medication; Chronic cardiac disease (not hypertension); Blood ph; Blood albumin; Urea nitrogen; LDH; Oxygen saturation and oxygen saturation measure on room air or oxygen therapy	0.81 (0.77 – 0.85)	NA	77	71	41	93
				XGBoost		0.82 (0.79 – 0.85)	NA	67	75	44	91
Pan D et al. 2020[32]	China	9 Feb – 10 Mar	**204** (**D:** 120 **V:** 84)	Multivariate logistic regression	CRP; PaO_2/FIO_2 and troponin I	0.988 (0.972 – 1.000)	NA	NA	NA	NA	NA
Pan P et al. 2020[33]	China	2 Feb – 15 Apr	**123** (**D:** 98 **V:** 25)	Log regression	LDH; prothrombin time; creatinine; lymphocytes; neutrophils; eosinophils; total bilirubin and albumin	0.84 (0.656 - 1)	0.3962	73.3	80	84.6	66.7
				AdaBoost		0,911 (0.802 – 1)	0.4283	66.7	90	90.9	64.3
				GBDT		0.85 (0.699 – 1)	0.4583	73.3	80	84.6	66.7
				XGBoost model		0.92 (0.814 – 1)	0.4478	80%	90	92.3	75
				CatBoost		0.9133 (0.799 – 1)	0.5063	86.7	80	86.7	80
Park JG et al. 2020[34]	Korea	19 Jan - 2 Mar	**289**	Cox regression	Age; AST; ALT and platelets	0.702 (0.627 – 0.778)	≥4.95	48.5	87.6	55%	84.4
	**Total Score: age × AST/ (platelet count × √ALT)**										

Author, Year[reference]	Country	Study period (2020)	N	Methods used to derivate nomograms	Predictors in final model	AUROC (95% confidence interval)	Cut-off value	Sensitivity (%)	Specificity (%)	Positive predictive value (%)	Negative predictive value (%)
Pfeifer N et al. 2022[73]	Germany	1 Mar – 31 Mar	**164**	Multivariate logistic regression	Age; Cognitive deterioration; O2 saturation; Lymphocytopenia; renal function deterioration; CRP; Monocyte count and Comorbidity	0.937	NA	NA	NA	NA	NA
Rahman T et al. 2021[74]	China	10 Jan - 18 Feb	**375**<br>**D:** 70%<br>**V:** 30%	Multivariate logistic regression	Age; Neutrophils count; Platelet count; Monocytes; WBC; Lymphocyte count and RDW	0.961	<10.8 (Low Risk (<5%)<br>10.8 – 10.96 (Moderate Risk (5 -50%)<br>> 10.96 (High Risk (>50%)	87	90	NA	NA
	**Total Score: -12.75911 + [0.5784669 x Neutrophils count (10^3/μL)] + (0.0724752 x Age) – [0.009611 x Platelet count (10^3/μL)] + [0.0931182 x Monocytes (%)] + [0.0064276 x WBC (10^3/μL)] – [3.567051 x Lymphocyte count (10^3/μL)] + [0.7140086 x RDW (%)]**										
Rahman T et al. 2021[75]	USA and China	**D:** 24 Mar – 30 Apr<br>**V:** 10 Jan – 18 Feb	**759**<br>**D:** 384<br>**V:** 375	Multivariate logistic regression	Age; Lymphocyte count; D-dimer; CRP and creatinine	**D:** 0.940<br>**V:** 0.987	<16.6 (Low Risk (<5%)<br>16.7 – 19.8 (Moderate Risk (5 -50%)<br>> 19.8 (High Risk (>50%)	91	78	NA	NA
	**Total Score: -0.7606855 + (1.904726 * Age) – (1.964625* Lymphocyte count) – (1.508334 * D-dimer) + (0.709297 * CRP) + (0.2467726 * Creatinine)**										
Salto-Alejandre S et al. 2020[35]	Spain	21 Feb – 8 Apr	**244**	Multivariate logistic regression	Peripheral capillary oxygen saturation; neutrophils; platelets; LDH and CRP	0.891 (0.847 – 0.936)	50%	62.5	90.1	68.6	87.4
	**Total Score: = –4.655 + 3.075 (SpO2 <95%) + 1.324 (neutrophil count >7.5 × 109 per L) + 1.492 (platelet count <130 × 109 per L) + 0.981 (LDH ≥300 UI/L) + 0.916 (CRP ≥100 mg/L)**										
Sánchez-Montañes M et al. 2020[76]	Spain	5 Feb – 23 Apr	**1696**	Logistic Regression<br>Decision Tree<br>RF<br>Tree Naive Bayes	Age; Gender; O2 Saturation; Residential Institution and Oncological Patient Deterioration	0.89<br>0.81<br>0.90<br>0.87	NA	80<br>73<br>87<br>79	83<br>83<br>79<br>79	46<br>45<br>44<br>41	96<br>94<br>97<br>95
Santos-Lozano A et al. 2020[36]	Spain	28 Feb – 30 Mar	**1369**	artificial neural network	Age; glomerular filtration rate; haemoglobin; albumin and LDH	0.91 (0.71–0.96)	NA	61	95	NA	NA
Soto-Mota A et al. 2020[16]	Mexico	30 Apr- 20 May	**400**	Multivariate logistic regression	Lymphocytes; Sat O2; white blood cells; hypertension; age; renal injury and myocardial injury	0,96 (0,94 - 0,98)	>65	63	97.5	96	72.5
	**Total Score: (X /(1+X) *100): Age (<40 (0,002) / 40 - 49 (0,004) / 50 - 59 (0,013) / 60 - 69 (0,037) / 70 - 79 (0,087) / >=80 (0,174) * HTA (x 2,06) * SO_2<88% (x 6,85) * Mio Injury (x 6) * WBC >10000 (x 4,23) *Lymphocytes <800 (x 2,89) * Renal Injury (x 4,23)**<br>https://lowharmcalc.com/										

Author, Year[reference]	Country	Study period (2020)	N	Methods used to derivate nomograms	Predictors in final model	AUROC (95% confidence interval)	Cut-off value	Sensitivity (%)	Specificity (%)	Positive predictive value (%)	Negative predictive value (%)
Torres-Macho J et al. 2020[50]	Spain	2 Mar - 31 May	**1968** (**D:** 1181 **V:** 393)	WoE Method<br>Gradient Boosting<br>Random Forest<br>SVM Linear Kernel<br>Neural Network<br>SVM Quadratic Kernel	Age; 0_2 saturation; creatinine; lymphocytes; haemoglobin; smoking; CRP; platelets and sodium	0.865<br>0.975<br>1<br>0.869<br>0.929<br>0.921	NA	NA	NA	NA	NA
	**Total Score: Age [<46 (-46) / 46-60 (4) / 61 – 78 (29) >78 (50) missing (28)] + O_2 Saturation [<88 (59) / 88–92 (36) / 93-96 (21) / ≥97 (-2) / missing (30)] + Creatinine [<0.77 (14) / 0.77-1.08 (21) / >1.71 (48) / missing (29)] + Lymphocytes [<500 (55) / 500-799 (37) / 800-100 (22) / missing (19)] + Hemoglobin [<11.2 (51) / 11.2-12.2 (36) / >12.2 (25) / missing (20)] + smoking [No (25) / Yes (43) / missing (22)] + CRP [<9.7 (8) / 9.7-65.9 (20) / 66-188.5 (35) / >188.5 (45) / missing (18)] + Platelets [<124 (48) / >124 (27) / missing (18)] + Sodium [<144 (28) / >144 (53) / missing (17)]** **www.pandemyc-score.co / m**										
Turcotte JJ et al. 2020[40]	USA	1 Mar – 12 Apr	**117**	logistic regression and Cox regression	Temperature at admission; supplemental O2 at admission; sputum production; insulin dependent diabetes and chronic kidney disease.	0.88 (0.812 – 0.956)	26.3*	83.7*	79.4*	NA	NA
	**Total Score: 22.316 – 0.242 temp + 0.189 O2L + 1.907 sputum + 2.471 IDDM + 1.567 CKD**										
Vaid A et al. 2020[38]	USA	15 Mar – 22 May	**4098** (**D:** 1514 **V:** 2584)	XGBoost<br>Log Regression<br>LASSO	Age; anion gap; CRP; LDH; oxygen saturation; BUN; ferritin; RDW; diastolic blood pressure and lactate (mortality at 7 days)	0.853 (0.85 – 0.856)<br>0.821 (0.818 – 0.824)<br>0.821 (0.818 – 0.823)	NA	50<br>46.3<br>46.2	94.4<br>92<br>92	NA<br>NA<br>NA	NA<br>NA<br>NA
Varol Y et al. 2020[39]	Turkey	11 Mar - 15 May	**383**	Logistic regression	Charlson comorbidity index; lymphocyte ratio; age and dyspnoea	0.802 (0.777 - 0.886)	2.5 poi nts	82	73	NA	NA
	**Total Score: Lymphocytes % [≥17.6 (0) / <17.6 (1)] + Age [<50 (0) / 50-65 (1) / ≥65 (2)] + CCI score [<3 (0) / ≥3 (1)] + Dyspnoea [Without (0) / With (1)]**										
Wang B et al. 2020[40]	China	Befo re 20 Mar	**104**	Logistic regression	Age; chest tightness (oppression); AST and BUN	0.893 (0.807 - 0.980)	NA	96	74.1	NA	NA

Author, Year[reference]	Country	Study period (2020)	N	Methods used to derivate nomograms	Predictors in final model	AUROC (95% confidence interval)	Cut-off value	Sensitivity (%)	Specificity (%)	Positive predictive value (%)	Negative predictive value (%)
Wang H et al. 2021[77]	China	1 Mar – 16 Apr	**4711**<br>**D:** 75%<br>**V:**25%	Multivariate models	Age; SpO2; MAP and Temperature	**D:** 0.798 (0.779 – 0.816)<br>**V:** 0.783 (0.751 – 0.815)	NA	80.4<br>80	65.6<br>61.6	NA<br>NA	NA<br>NA
	**Total Score: 0.742 + (0.006 x Age) + (-0.007 x SpO_2) + (0.012 x Temperature) + (-0.008 x MAP)**<br>**https://wanghai.shinyapps.io/dynnomapp/**										
Wang J et al. 2020[14]	China	28 Jan - 4 Mar	**278**<br>(**D:** 234<br>**V:** 44)	Multivariate logistic regression	Age; ferritin and D-dimer	0.871	≥85	86.4	81.8	39.6	91.7
	**Total Score: age + (0.01X ferritin (μg/L) +D-dimer (μg/mL)**										
Wang K et al. 2020[78]	China	**D:** 7 Jan – 11 Feb<br>**V:** 1 Feb – 20 Feb	**340**<br>**D:** 296<br>**V:** 44	Multivariate logistic regression and XGBoost / AIC	**Clinical model:** Age; Hypertension and Coronary heart disease<br>**Laboratory model:** Age; SpO2; Neutrophils count; Lymphocyte count; hsCRP; D-dimer; AST and GFR	0.88 (0.80 – 0.95)<br>0.83 (0.68 – 0.93)	NA	92.3<br>64.3	77.4<br>93.3	21.4<br>81.8	99.3<br>84.8
Wang R et al. 2020[41]	China	Jan - Feb	**450**	Multivariate logistic regression	WBC; prognostic nutritional index [serum albumin (g/L) + 5 ×lymphocyte count (10^9/L)] and LDH	0.950 (0.922 - 0.978)	NA	84.1	92.2	NA	NA
Weng Z et al. 2020[11]	China	1 Jan – 15 Feb	**301**<br>(**D:** 176<br>**V:** 125)	LASSO	Age; NLR and CRP	0.921 (0.835 – 0.968)	< 59<br>> 101	95.2<br>52.4	70.3<br>96.8	30.3<br>69.8	99.1<br>93.8
	**Total Score: (1.14 × age – 20) + (1.63 × NLR) + (5.00 × D – dimer) + (0.14 × CRP)**										
Wu G et al. 202[13]	China	16 Feb – 20 Mar	**110**	LASSO	Urea; WBC; prothrombin activity; IL-2R; indirect bilirubin; myoglobin and fibrinogen degradation products	0.997 (0.99 - 1.00)	NA	98	91	NA	NA
Xiao F et al. 2021[79]	China	Jan – Feb	**74**	Cox regression and LASSO	Age; Malignancy; Highest temperature; Radiomics lesion and Radiomics lung	C- Statistic 0.899 (0.850 – 0.950)	NA	NA	NA	NA	NA
Xiao LS et al. 2020[42]	China	1 Jan – 18 Mar	**442**<br>(**D:** 231<br>**V:** 211)	Multivariate logistic regression	Hypertension; Neutrophils; CRP; lymphocytes and LDH	0.861 (0.800 – 0.922)	>=-1.508	85	76	NA	NA
	**Total score: 1.035 x neutrophil count (1: 6.3 x 10^9/L; 0: <6.3 x 10^9/L) - 1.237 x lymphocyte count (1: 1.1 x 10^9/L; 0: <1.1 x 10^9/L) + 1.419 x CRP level (1: 10 mg/L; 0: <10 mg/L) + 1.066 x LDH (1: >245 U/L; 0: 245 U/L) + 0.969 x hypertension (1: with; 0: without) - 2.425**										

Author, Year[reference]	Country	Study period (2020)	N	Methods used to derivate nomograms	Predictors in final model	AUROC (95% confidence interval)	Cut-off value	Sensitivity (%)	Specificity (%)	Positive predictive value (%)	Negative predictive value (%)
Yadaw AS et al. 2020[43]	USA	9 Mar - 6 Apr	**3841** (**D:** 2880 **V:** 961)	Logistic regression; RF; SVM; XGBoost	Age; sex; ethnicity; encounter type; temperature; systolic blood pressure; diastolic blood pressure; oxygen saturation; lowest oxygen saturation; smoking; asthma; COPD; hypertension; obesity; diabetes; HIV and cancer	0.91 (0.88 - 0.94)	NA	NA	NA	NA	NA
Yan L et al. 2020[44]	China	10 Jan - 18 Feb	**485** (**D:** 375 **V:** 110)	XGBoost	LDH; lymphocytes and CRP	0.978	NA	NA	NA	NA	NA
Yu J et al. 2021[80]	China	24 Jan – 26 Apr	**314**	Cox regression	Age; admission classification; WBC; Neu%, Ferritin; CEA; and D-Dimer	C- Statistic 0.84 (0.79 – 0.88)	NA	NA	NA	NA	NA
Zhang B et al. 2020[40]	China	3 Jan – 20 Mar	**233**	LASSO	Age; LDH; AST; prothrombin time; serum creatinine; sodium; fasting blood glucose and D-dimer	0.943 (0.927 – 0.957)	0.190	96.8%	82.4%	58%	99%
Zhang S et al. 2020[45]	China	12 Jan - 9 Feb	**828**	Cox regression	Age; LDH; NLR and direct bilirubin	C- Statistic 0.886 (0.873–0.899)	NA	NA	NA	NA	NA
	**Total Score: [(Age-20) x1.25] + [NLR>8 (26.6) / NLR≤8 (0)] + [Direct bilirubin>5 (16.06) / Direct bilirubin≤5 (0)] + [LDH>360 (67.63) / LDH≤360 (0)]**										
Zhao Z et al. 2020[46]	USA	9 Mar – 20 Apr	**641** (**D:** 454 **V:** 187)	Logistic regression	Age; heart failure; procalcitonin; LDH; COPD; SpO_2 and heart rate	0.87 (0.83-0.92)	NA	7.1%	100%	NA	NA
	**Total Score: Heart Failure (1) + Procalcitonin >0.34 ng/mL (1) + LDH>460 U/L (1) + COPD (1) + SpO_2<92% (1) + Heart rate>117 bpm (1) + Age>63 years (1)**										
Zhou J et al. 2020[2]	China	12 Jan - 26 Feb	**118**	Multivariate logistic regression	LDH; BUN; D-dimer; albumin and NLR	0.955	0.6005	88.9%	98.4%	NA	NA
Zinellu A et al. 2020[47]	Italy	15 Mar - 15 May	**115**	Cox regression	AST and ALT	0.701 (0.603-0.787)	<1.49	74%	70%	NA	NA
	**Total score: AST / ALT**										

N: Participants for model development **(D)** or internal validation **(V)** sets. **ACE-inhibitor:** Angiotensin converting enzyme inhibitor; **ALT:** Alanine aminotransferase; **ANN:** artificial neural network; **AR blocker:** Angiotensin II receptor blocker; **AST:** Aspartate aminotransferase; **BUN**: Blood urea nitrogen; **CEA:** Carcioembryonic antigen; **CKD:** Chronic kidney disease; **COPD:** Chronic obstructive pulmonary disease; **CRP:** C-reactive protein; **cTnI:** cardiac troponin I; **D-Bil:** Direct bilirubin; **DM:** Diabetes mellitus; **DPP-4 inhibitor:** Dipeptidyl peptidase 4 inhibitor; **GBDT**: gradient boosted decision tree; **GFR:** glomerular filtration rate; **IL-2R:** Interleukin 2 receptor; **IL-6:** Interleukin 6; **KNN:** k-nearest neighbours; **LASSO**: least absolute shrinkage and selection operator; **LDH:** Lactate dehydrogenase; **MAP:** mean arterial pressure; **NA:** Not available**; Neu%**: neutrophils percentage; **NLR:** Neutrophils to lymphocyte ratio; **NOD**: number of days of symptoms prior to hospital admission; **NSAID:** Nonsteroidal anti-inflammatory drugs; **P/F ratio:** arterial oxygen pressure (PaO2) / fractional inspired oxygen (FiO2); **PLS:** Partial least square; **RBF-SVM:** SVM with radial basis function kernel; **RDW:** Red cell distribution width; **RF:** random forest; **SLE:** Systematic lupus erythematosus; **SVM**: support vector machine; **WBC:** White blood cells. *optimal cut-off for ICU admission or death.
