# Supplementary material for: Predicting In-Hospital Mortality in Severe COVID-19: A Systematic Review and External Validation of Clinical Prediction Rules
Source: Biomedicines. 2022 Sep 27;10(10):2414. doi: 10.3390/biomedicines10102414 (PMC9599062; doi:10.3390/biomedicines10102414)
Supplement: Supplementary file 1 [file biomedicines-10-02414-s001.zip › Supplementary Table S3.pdf]

Supplementary Table S3. Risk of bias assessment (using PROBAST) based on four domains across the studies that developed and/or validated prediction models for in-hospital mortality due to coronavirus disease 2019

Study (author, journal, year)[reference]	Type of model	ROB				Applicability			Overall	
		Participants	Predictors	Outcome	Analysis	Participants	Predictors	Outcome	ROB	Applicability
Abdulaal A. BMC Med Inform Decis Mak 2020[18]	Development and validation	+	+	+	?	+	+	+	?	+
Acar HC. BMC Infect Dis 2020[56]	Development	+	+	+	-	+	+	+	-	+
An C. Sci Rep 2020[19]	Development and validation	+	+	+	?	-	+	+	?	-
Bellan M. Dis Markers 2021[57]	Development	+	?	+	-	+	+	+	-	+
Bello-Chavolla OY. J Clin Endocrinol Metab 2020[49]	Development	?	?	+	-	+	+	+	-	+
Besutti G. Eur Radiol 2021[58]	Development	+	?	+	-	+	+	+	-	+
Chen B. Comput Struct Biotechnol J 2021[59]	Development and validation	+	+	+	+	+	+	+	+	+
Chen H. J Med Virol 2020[20]	Development	+	+	+	+	+	+	+	+	+
Chen R. Chest. 2020[21]	Development	+	+	+	+	+	+	+	+	+
Cheng A. Int J Antimicrob Agents 2020[22]	Development	+	?	+	+	+	+	+	?	+
Chowdhury MEH. Cognit Comput 2020[60]	Development	+	?	+	-	+	+	+	-	+
Clift AK. BMJ 2020[23]	Development and validation	?	+	+	+	?	+	+	?	?
Ding Z-y. J Hepatol 2021[54]	Development	+	+	+	-	+	+	+	-	+
Dong YM. Clin Infect Dis 2021[61]	Development and validation	+	+	+	+	+	?	+	+	?
Ebell MH. J Am Board Fam Med 2021[62]	Development and validation	+	+	+	+	+	+	+	+	+
Foieni F. Intern Emerg Med 2020[17]	Development and validation	-	+	+	?	-	+	?	-	-
Gao Y. Nat Commun 2020[24]	Development and validation	+	+	+	+	+	+	+	+	+
Guan X. Ann Med 2021[63]	Development and validation	+	?	+	+	-	+	+	?	-
Harmouch F. J Med Virol 2020[64]	Development	+	+	+	-	+	+	+	-	+
He J. Front Med (Lausanne) 2021[65]	Development	+	+	+	+	-	+	+	+	-
Hu C. Int J Epidemiol 2020[48]	Development and validation	?	+	+	-	+	+	+	-	+
Ji D. Clin Infect Dis. 2020[12]	Development	-	+	+	+	-	+	+	-	-
Jian M. Ann Transl Med 2021[66]	Development and validation	+	?	+	?	+	+	+	?	-
Kamran SM. Cureos. 2020[25]	Development	?	+	+	-	-	+	?	-	-
Kim DH. Medicine (Baltimore) 2021[68]	Development	?	?	+	-	+	+	+	-	+
Ke Z. Acta Radiol 2022[67]	Development and validation	?	?	+	+	+	+	+	?	+
Knight SR. BMJ. 2020[15]	Development and validation	+	+	?	+	+	+	+	?	+
Li J. Ann Palliat Med. 2021[69]	Development	+	?	+	-	+	+	+	-	+
Li L. Aging 2021[70]	Development and validation	+	?	+	?	-	+	+	?	-
Liang W. JAMA Intern Med 2020[26]	Development and validation	+	?	+	+	?	?	?	?	?
Liu J. Clin Respir J. 2020[27]	Development	+	+	+	+	?	+	?	+	?
Liu Q. Risk Manag Healthc Policy. 2021[51]	Development and validation	+	+	+	+	+	+	+	+	+

Lorente L. Anaesth Crit Care Pain Med 2020[28]	Development	-	+	?	-	-	-	+	-	-
Ma X. Front Public Health 2020[29]	Development	+	+	?	+	+	?	?	?	?
Ma X. Epidemiol Infect 2020[30]	Development and validation	+	+	+	+	+	+	+	+	+
Ma X. BMC Infect Dis 2020[31]	Development	+	+	+	+	+	+	+	+	+
Magro B. PLoS One 2021[52]	Development and validation	?	+	+	?	+	+	+	?	+
Moon HJ. J Korean Med Sci. 2021[71]	Development and validation	+	+	+	+	-	+	+	+	-
Niu Y. Disaster Med Public Health Prep. 2021[53]	Development and validation	+	+	+	+	+	+	+	+	+
Ottenhoff MC. BMJ Open 2021[72]	Development and validation	+	+	+	?	+	+	+	?	+
Pan D. Front Public Health 2020[32]	Development and validation	+	+	+	+	+	+	+	+	+
Pan P. J Med Internet Res 2020[33]	Development and validation	-	+	?	+	-	+	?	-	-
Park JG. BMJ Open. 2020[34]	Development	+	+	+	-	+	+	+	-	+
Pfeifer N. Med Klin Intensivmed Notfmed 2022[73]	Development	+	?	+	-	+	?	+	-	?
Rahman T. Diagnostics. 2021[74]	Development	+	+	+	-	+	+	+	-	+
Rahman T. IEEE Access. 2021[75]	Development and validation	+	?	+	+	-	?	+	?	-
Salto-Alejandre S. J Infection. 2020[35]	Development	+	+	?	+	?	+	-	?	-
Sánchez-Montañés M. Int J Environ Res Public Health 2020[76]	Development and validation	+	?	+	+	+	+	+	?	+
Santos-Lozano A. Clin Chem Lab Med 2020[36]	Development	+	+	+	-	+	+	+	-	+
Soto-Mota A. J Am Coll Emerg Physicians Open 2020[16]	Development	+	+	+	-	+	+	+	+	-
Torres-Macho. J Clin Med 2020[50]	Development and validation	+	+	+	+	+	+	+	+	+
Turcotte JJ. PLoS One. 2020[40]	Development	+	+	+	-	?	+	?	-	?
Vaid A. J Med Internet Res 2020[38]	Development and validation	+	+	+	+	?	+	?	+	?
Varol Y. Int J Clin Pract. 2020[39]	Development	+	+	+	-	+	+	+	-	+
Wang B. Jpn J Infect Dis 2020[40]	Development	+	+	+	?	?	+	+	?	?
Wang H. Front Med (Lausanne) 2021[77]	Development and validation	+	?	+	+	+	+	+	?	+
Wang J. J Int Med Res. 2020[14]	Development and validation	+	+	+	?	+	+	+	?	+
Wang K. Clin Infect Dis 2020[78]	Development and validation	+	+	+	+	-	+	+	+	-
Wang R. J Clin Lab Anal 2020[41]	Development	+	-	+	+	+	¿	+	-	+
Weng Z. J Transl Med 2020[11]	Development and validation	+	+	+	+	+	+	+	+	+
Wu G. Sci Rep 2020[42]	Development	+	+	+	+	+	+	+	+	+
Xiao F. Phys Med Biol 2021[79]	Development	?	+	-	-	+	+	+	-	+
Xiao LS. EBioMedicine 2020[13]	Development and validation	?	+	?	+	?	+	?	?	?
Yadaw AS. Lancet Digit Health 2020[43]	Development and validation	+	+	+	+	?	+	?	+	?
Yan L. Nat Mach Intell 2020[44]	Development and validation	+	+	+	+	+	+	+	+	+
Yu J. Front Med (Lausanne) 2021[80]	Development	+	+	?	-	-	+	?	-	-
Zhang B. Fr ont Med 2020[40]	Development and validation	+	+	?	+	?	+	?	?	?
Zhang S. Critical Care 2020[45]	Development and validation	+	+	+	+	+	+	+	+	+

Zhao Z. PLoS One. 2020[46]	Development and validation	?	?	?	+	?	?	?	?	?
Zhou J. Current Med Res Opin 2020[2]	Development	+	+	+	+	+	+	+	+	+
Zinellu A. Eur J Clin Invest 2020[47]	Development	+	-	+	-	-	+	+	-	+

PROBAST = Prediction model Risk Of Bias ASsessment Tool; ROB = risk of bias.
* + indicates low ROB/low concern regarding applicability; − indicates high ROB/high concern regarding applicability; and ? indicates unclear ROB/unclear concern regarding applicability.
